# Ultrahigh Carrier Mobilities in Ferroelectric Domain Wall Corbino Cones at Room Temperature

**DOI:** 10.1002/adma.202204298

**Published:** 2022-07-11

**Authors:** Conor J. McCluskey, Matthew G. Colbear, James P. V. McConville, Shane J. McCartan, Jesi R. Maguire, Michele Conroy, Kalani Moore, Alan Harvey, Felix Trier, Ursel Bangert, Alexei Gruverman, Manuel Bibes, Amit Kumar, Raymond G. P. McQuaid, J. Marty Gregg

**Affiliations:** ^1^ School of Mathematics and Physics Queen's University Belfast Belfast BT7 1NN UK; ^2^ Department of Physics & Bernal Institute University of Limerick Limerick V94 T9PX Ireland; ^3^ Unité Mixte de Physique CNRS Thales Université Paris‐Saclay Palaiseau 91767 France; ^4^ Department of Energy Conversion and Storage Technical University of Denmark Kongens Lyngby 2800 Denmark; ^5^ Department of Physics and Astronomy University of Nebraska Lincoln NE 68588 USA; ^6^ Present address: Department of Materials Imperial College London Royal School of Mines Exhibition Road London SW7 2AZ UK

**Keywords:** carrier mobility, Corbino disks, domain walls, ferroelectrics, magnetoresistance

## Abstract

Recently, electrically conducting heterointerfaces between dissimilar band insulators (such as lanthanum aluminate and strontium titanate) have attracted considerable research interest. Charge transport and fundamental aspects of conduction have been thoroughly explored. Perhaps surprisingly, similar studies on conceptually much simpler conducting homointerfaces, such as domain walls, are not nearly so well developed. Addressing this disparity, magnetoresistance is herein reported in approximately conical 180° charged domain walls, in partially switched ferroelectric thin‐film single‐crystal lithium niobate. This system is ideal for such measurements: first, the conductivity difference between domains and domain walls is unusually large (a factor of 10^13^) and hence currents driven through the thin film, between planar top and bottom electrodes, are overwhelmingly channeled along the walls; second, when electrical contact is made to the top and bottom of the domain walls and a magnetic field is applied along their cone axes, then the test geometry mirrors that of a Corbino disk: a textbook arrangement for geometric magnetoresistance measurement. Data imply carriers with extremely high room‐temperature Hall mobilities of up to ≈3700 cm^2^ V^−1^ s^−1^. This is an unparalleled value for oxide interfaces (and for bulk oxides) comparable to mobilities in other systems seen at cryogenic, rather than at room, temperature.

## Graphical abstract

Magnetoresistance is measured in conical 180° charged domain walls, in ferroelectric lithium niobate. Extracted carrier mobilities are extremely high, up to ≈3700 cm^2^ V^−1^ s^−1^. This is an unparalleled value for oxide interfaces (and for bulk oxides too) and is most comparable to mobilities in other systems typically seen at cryogenic, rather than at room, temperature.

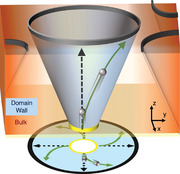

## 1 Introduction

The idea that two adjoining electrically insulating materials might generate a new sheet‐like conducting state, along the interface between them, is remarkable. However, it is not new. Almost 50 years ago, in a theoretical study, Vul et al.^[^ ^1^ ^]^ considered boundaries between two insulating dielectrics, with opposing senses of spontaneous electrical polarization; a local accumulation of charge was postulated of sufficient density that metallic interfacial behavior was expected. Experimentally, signs of domain wall conduction were later seen (e.g., ref. [2]), and in the 1999s, Aird and Salje^[^ ^3^ ^]^ even reported superconductivity along specific twin boundaries (in tungsten trioxide). Somehow, these studies failed to prompt immediate followup activity from the rest of the scientific community. It was only in 2004, when Ohtomo and Hwang reported distinct electrical conductivity at interfaces between single‐crystal SrTiO_3_ substrates and LaAlO_3_ thin films^[^ ^4^ ^]^ that the field really exploded. Since then, considerable research has been done and a great deal is now known about the fundamental physics of conduction, along oxide heterointerfaces (those between two different band insulators) in particular.

Homointerfaces (boundaries between distinct regions of the same insulating material) have not been ignored, but the nature of research has been somewhat different. For ferroelectric domain walls, in particular, there has been a phase of simply discovering new systems in which enhanced electrical conduction can been seen in both proper (BiFeO_3_,^[^ ^5^, ^6^ ^]^ lithium niobate (LiNbO_3_, LNO),^[^ ^7^, ^8^ ^]^ BaTiO_3_,^[^ ^9^ ^]^ and Pb(Zr*_x_* Ti_1−_ *_x_*)O_3_^[^ ^10^ ^]^) and improper systems (hexagonal manganites,^[^ ^11^ ^]^ copper chlorine boracites,^[^ ^12^ ^]^ and Ruddlesden–Popper phases^[^ ^13^ ^]^). Significant focus has also been given to the manipulation of domain walls; their site‐specific controlled injection, removal, and movement have been established,^[^ ^12^, ^14^, ^15^, ^16^, ^17^, ^18^ ^]^ and conductance states have been dynamically modified (by tuning their number^[^ ^19^ ^]^ and inclination angles^[^ ^20^, ^21^ ^]^). A primary driver for all of this research has been the possibility of combining 2D electrical conduction with an inherently dynamic nature (domain walls can be created, moved, and destroyed by externally applied fields) to realize a new paradigm of reconfigurable “domain wall electronics”:^[^ ^22]^ a technology in which domain wall nanocircuitry might be formed, changed, and erased on demand. This exciting idea has already motivated the demonstration of a number of different domain wall devices such as bit memories, memristors, and transistors.^[^ ^19^, ^23^, ^24^, ^25^, ^26^, ^27^ ^]^

Fundamental explorations have also been done: evidence has been found for defect aggregation producing interband dopant states at ferroelectric domain walls;^[^ ^28^ ^]^ bending of the electronic bands due to polar discontinuities has been postulated;^[^ ^29^ ^]^ and alterations in the electronic band structure have been seen to produce a slight narrowing of the bandgap over that established in bulk.^[^ ^5^, ^11^, ^30^ ^]^ Quantitative aspects of transport behavior, based on scanning‐probe‐microscopy‐enabled Hall voltage measurements, have demonstrated relatively high carrier mobilities (of the order of hundreds of cm^2^ V^−1^ s^−1^)^[^ ^31^, ^32^ ^]^ in both ErMnO_3_ and YbMnO_3_. Rather recently, machine‐learning‐assisted data analysis has also been employed, untangling the domain wall response from bulk and interface effects.^[^ ^33^ ^]^

Taken as a whole, however, insight into the fundamental physics of transport in homointerfacial ferroelectric domain walls is distinctly underdeveloped and has been somewhat in the shadow of the technology‐driven work alluded to above. This is particularly evident when progress is compared to the state‐of‐the‐art in heterointerfacial oxide boundary research.

Here, we seek to redress this imbalance by reporting magnetoresistance (MR) measurements on charged conducting domain walls, created by partial switching, in ion‐sliced single‐crystal LiNbO_3_ thin films (500 nm thick). The domain walls are approximately conical in morphology, and we show that this allows magnetotransport to be interpreted in terms of the classical Corbino disk geometry,^[^ ^34^, ^35^, ^36^, ^37^ ^]^ which is distinct from the magnetoresistance seen previously at conducting domain walls in BiFeO_3_.^[^ ^38^, ^39^ ^]^ Our geometric magnetoresistance measurements imply an exceptionally high‐room‐temperature domain wall Hall mobility, of the order of thousands of cm^2^ V^−1^ s^−1^. This is dramatically larger than reported in other oxide semiconductors^[^ ^40^, ^41^, ^42^ ^]^ and heterointerfaces,^[^ ^4^, ^43^ ^]^ where the highest room‐temperature mobilities observed are in the region of tens to hundreds of cm^2^ V^−1^ s^−1^. Transport comparisons are perhaps better made with intrinsic elemental semiconductors (Si = 1350 cm^2^ V^−1^ s^−1^, Ge = 3600 cm^2^ V^−1^ s^−1^)^[^ ^44^ ^]^ or 2D electron gases at low temperatures. We note, however, that hints of extremely high carrier mobilities in LiNbO_3_ have been seen previously, in photocurrent experiments.^[^ ^45^, ^46^ ^]^ Our observations, while dramatic, are therefore not completely without precedent. Nevertheless, they do suggest extraordinary transport behavior in these domain walls. In addition to mobility, we also infer the carrier density to be a small fraction (10^−5^) of that required for screening the polar discontinuity at the wall, and this indicates, as has been seen in other domain wall systems,^[^ ^31^, ^32^ ^]^ that the overwhelming majority of assumed screening charges do not participate in conduction.

## 2 LNO Domain Wall Morphology

**Figure 1:**
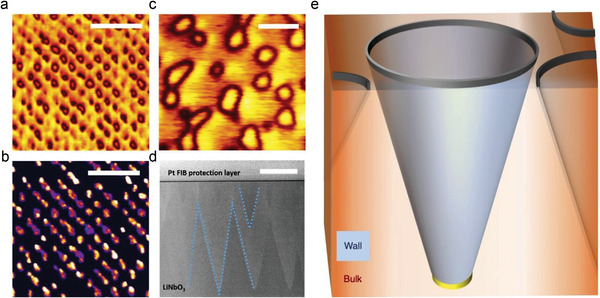
Conducting, conical domain walls in LiNbO_3_. a) Piezoresponse force microscopy (PFM) amplitude and b) conducting atomic force microscopy (cAFM) maps of domains obtained on the top surface of 500 nm‐thick partially switched lithium niobate. Domains were created using a rastered AFM tip as a top electrode. Scale bar: 700 nm. c) PFM amplitude from the top surface of a similar LiNbO_3_ (LNO) thin film sample after partial switching using a mesoscale liquid top electrode. Scale bar: 150 nm. d) Cross‐sectional high‐angle annular dark‐field scanning transmission electron microscopy (HAADF‐STEM) image of the domains in LNO. The overlaid lines highlight the inclination of the walls. Scale bar: 200 nm. e) A schematic of the conducting domain wall cones in LNO, as inferred from the top surface PFM in (a) and (c), and the cross‐sectional HAADF‐STEM in (d). The dark gray and yellow rings highlight the locus of the electrical contacts made with the top and bottom electrodes, respectively.

Our studies were performed on commercially obtained 500 nm thick ion‐cut single‐crystal thin films, with 150 nm thin‐film gold–chromium bottom electrodes. The uniaxial polarization in these films is perpendicular to their surfaces (*z*‐cut). As received, the LNO was in a monodomain state. However, partial switching of polarization, under an applied electric field (using an additional top electrode), caused needle‐like domains to grow. After traversing the interelectrode gap, needle domains grow radially outward, maintaining a rather strong inclination with respect to the polar axis. This results in a truncated cone morphology across the interelectrode gap. **Figure** **1**a shows the circular base sections (dark contrast) of a number of domain‐wall‐truncated cones, evident on the top surface of an LNO film, along with associated electrical conduction footprints (Figure 1b). Note that current maps are somewhat smeared, but this does not indicate interelectrode conduction through domains themselves. It instead reflects the finite size of the conducting atomic force microscopy (AFM) tip (radius ≈ 20–40 nm), maintaining electrical contact with domain walls, even when the center of the tip has moved past them, as well as the possibility of low‐resistance pathways that involve traversing a short section of LNO at the surface, to reach strongly conducting domain wall source–drain conduits. Piezoresponse force microscopy (PFM) and conducting AFM (cAFM) images in literature (for example, in ref. [19]) show similar smearing effects, but scans using new sharp tips systematically reveal that conduction arises at the domain wall (see Section S10 in the Supporting Information). While these specific domain patterns were written using a raster‐scanned AFM tip as the top electrode (a sharp‐point field source), we observed a similar microstructure, even when poling with a liquid metal indium–gallium–tin eutectic mesoscale planar top electrode (Figure 1c). Conical structures were confirmed in cross section by the appearance of domain contrast in approximately isosceles triangular forms (Figure 1d). The microstructure, which we have schematically represented in Figure 1e, is consistent with various other domain investigations across both bulk and thin‐film LNO samples.^[^ ^19^, ^20^, ^24^, ^47^ ^]^

## 3 LNO Domain Walls as Corbino Cones

Current travelling through a solid is deflected by a magnetic field, due to the magnetic component of the Lorentz force. The consequences of this deflection depend on the sample geometry. In a Hall bar, for example, carriers are deflected toward insulating sample edges, where they accumulate, to develop an electric field, which builds to counteract the magnetic‐field‐induced deflection. Ideally, in equilibrium, carriers return to their straight‐line path along the external electric field direction, and a Hall potential is maintained on the transverse sample boundaries. If no insulating sample boundaries exist, then no Hall field can develop, the magnetic deflection of the current persists, and carriers deviate from their shortest path between source and sink electrodes. This increase in carrier path length leads to a magnetic‐field‐dependent increase in the sample resistance, termed geometric magnetoresistance.^[^ ^35^ ^]^ The magnitude of the magnetic force on the moving carrier is governed by its drift velocity in the electric field. Therefore, the size of the magnetic deflection, and resulting resistance change, can be used to infer carrier mobility.^[^ ^48^, ^49^, ^50^ ^]^ This approach is commonly used to characterize the mobility active in short‐channel device geometries, where the sample is much wider than it is long^[^ ^51^, ^52^, ^53^, ^54^, ^55^ ^]^ (*l*/*w* ≪ 1, where *l* is the length between current carrying electrodes and *w* is the channel width). In this geometry, known as the “short Hall bar,” many carriers, which are deflected by a Lorentz force, reach the sink electrode before encountering a sample boundary. Thus, the full Hall potential does not develop, and carrier deflection persists. Intermediate geometries have also been studied:^[^ ^56^, ^57^, ^58^ ^]^ in rectangular samples, where *l*/*w* < 3, some Hall potential is maintained on the sample boundaries, but it is insufficient to fully counteract the magnetically induced deflection, and a (reduced) MR persists.

**Figure 2:**
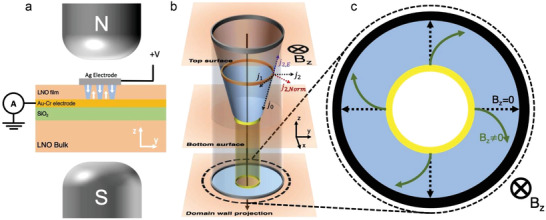
Domain walls as Corbino disks. a) Experimental setup for the geometric magnetoresistance measurement. b) Schematic depicting conducting domain wall in LNO, along with its 2D projection, which takes the form of the Corbino disk. The black arrow shows the direction of the magnetic field used in the geometric MR measurement depicted in (a), and the various dotted arrows show the current components found through an iterative description of the current density. c) The typical Corbino disk geometry, employed for geometric magnetoresistance measurements. It consists of a sample under investigation (blue annulus) and concentric inner and outer electrodes (black and yellow rings). Electronic motion in the presence and absence of a perpendicular magnetic field is indicated by full green and dashed black arrows, respectively.

While the ideal Hall bar (*l*/*w* = ∞) is not realizable, the ideal geometric magnetoresistor is. It is the well‐known Corbino disk^[^ ^35^, ^56^ ^]^ geometry (**Figure** **2**c): an annular sample sandwiched between two concentric electrodes. This geometry is the limiting case of the short Hall bar (*l*/*w* = 0). It completely mitigates any carrier buildup, as the only boundaries present are at the electrodes. No Hall field develops and a full geometric magnetoresistance is manifested; Hall fields are effectively “shorted.”

The equivalence of the conical conducting domain walls, connecting top and bottom electrodes, and the Corbino disk geometry can be loosely inferred by geometric projection (onto the plane parallel to the LNO sample surface); the conical conducting LNO domain wall is then the Corbino annulus, and the domain wall intersections with the top and bottom electrodes form the concentric outer and inner contacts (black and yellow rings in Figure 1e), illustrated schematically in Figure 2b,c. It is important, however, to explicitly derive the form of the magnetoresistance response for the cone.

Typically, geometric magnetoresistance varies quadratically with the applied magnetic field.^[^ ^35^ ^]^ This conclusion can be drawn from the analytic solution for the full current density, in the presence of magnetic and electric fields (***B*** and ***E***) in a Drude‐type model of conduction (Sections S1–S3, Supporting Information). A Hall field can be included, but only if its form is known. For the conical domain wall geometry, with no Hall correction considered, this analytic solution suggests that current pathways will deviate from the domain wall itself and into bulk LNO. Given its highly insulating nature, this is unphysical and so we conclude that a small Hall potential must develop across the width of the domain wall, creating a Hall field perpendicular to the domain wall surface, to confine the carriers inside the conducting conduit.

To accurately assess the full current density in the conical geometry, including an estimation of any Hall field, we use an iterative summation procedure. Here, we consider the action of the magnetic part of the Lorentz force on carriers as a series of successive, diminishing current components, which are corrections to the conventional current drift in an electric field, directed along the major axes in the system. Each successive correction is a higher order of the principal deflection *μB*, with the sum converging if *μB* < 1. This allows us to account for Hall fields by applying geometry‐specific boundary conditions: current components directed off the cone are cancelled by a Hall component, while current remaining on the cone is allowed, and this component forms the basis of the next iteration. This approach is taken from that presented in ref. [35]. Its applicability is discussed further in Sections S4 and S5 (Supporting Information), but the essence and implied form of the geometric magnetoresistance response is summarized in the following sections.

The electric and magnetic fields acting on the current‐carrying domain wall truncated cones can be represented in cylindrical coordinates as

(1) $$E_{0}=\left(\begin{array}{c} E_{r}\\ E_{\phi }\\ E_{z} \end{array}\right)=E_{0}\left(\begin{array}{c} -\cos\theta \\ 0\\ -\sin\theta \end{array}\right)$$

(2) $$B=\left(\begin{array}{c} B_{r}\\ B_{\varphi }\\ B_{z} \end{array}\right)=B_{0}\left(\begin{array}{c} 0\\ 0\\ -1 \end{array}\right)$$

where *r*, φ, and *z* represent the radial, azimuthal, and *z*‐axis bases of cylindrical coordinates, and θ is the inclination angle of the domain wall, defined as the acute angle between the domain wall surface normal and the *z*‐axis. *E*_0_ and *B*_0_ are the magnitudes of the electric and magnetic fields, respectively.

Walking through the first few iterations of this cycle reveals the key behavior. Initially, current moves along the electric field direction which defines the cone surface

(3) $$j_{0}=\sigma_{0}E_{0}$$

The first deflection is then along ***E***_**0**_ × ***B***, which is azimuthal in this case

(4) $$\begin{array}{c} j_{1}=\sigma_{0}E_{1}=\sigma_{0}(v_{d1}\times B)=\sigma_{0}\mu (E_{0}\times B)\\ =\mu (j_{0}\times B)=\sigma_{0}E_{0}\left(-\begin{array}{c} 0\\ \mu B_{0}\cos\theta \\ 0 \end{array}\right) \end{array}$$

Here, ***E***_**1**_ is an “effective electric field,” which would be that needed to generate the carrier deflection that is actually caused by the magnetic component of the Lorentz field, and ***v***_**d1**_ is the drift velocity due to this effective field. ***j***_**1**_ is azimuthal and remains on the cone, so no Hall correction is required yet.

The next deflection suffered by the carriers is radial

(5) $$j_{2}=\mu (j_{1}\times B)=\sigma_{0}E_{0}\text{​}\left(\begin{array}{c} \mu^{2}B_{0}^{2}\cos\theta \\ 0\\ 0 \end{array}\right)$$

and clearly does not remain on the cone surface (Figure 2b). Given that carriers cannot propagate into the highly insulating bulk LNO (as discussed above), this component illustrates that a Hall potential must form across the width of the domain wall itself. It is in this detail that the current behavior in the conical system differs from that in the true planar Corbino disk, while we still have an increase in path length due to the azimuthal component of Equation (4), and thus expect a geometric magnetoresistance; we also expect a small Hall potential, justifying the use of the iterative investigation and approach.

***j***_**2**_ gives information on the form of the magnetoresistance and Hall potential. Projecting ***j***_**2**_ onto the external electric field direction, we find a current component opposing the initial current direction (shown in purple in Figure 2b)

(6) $$j_{2,E}=\frac{j_{2}\cdot E_{0}}{|E_{0}{|}^{2}}E_{0}=\sigma_{0}E_{0}\left(\begin{array}{c} \mu^{2}B_{0}^{2}\cos^{3}\theta \\ 0\\ \mu^{2}B_{0}^{2}\sin\theta \cos^{2}\theta \end{array}\right)=-\mu^{2}B_{0}^{2}\cos^{2}\theta \sigma_{0}E_{0}$$

This is the first approximation of the magnetoresistance. Only this allowed current component is then used in the next series of iterations. The remaining component of ***j***_**2**_ (***j***_**2,Norm**_, red arrow in Figure 2b) is orientated along the cone surface normal and will first develop, and then be countered by a Hall potential, as outlined above. Following the procedure developed in Equations (3)–(6) through a few more iterations (shown in the Supporting Information), a full current density in the electric field direction is as follows

(7) $$j_{B,E}=(1-\mu^{2}B_{0}^{2}\cos^{2}\theta +\mu^{4}B_{0}^{4}\cos^{4}\theta +\cdot \cdot \cdot )\sigma_{0}E$$

This approximates to a full solution of

(8) $$j_{B,E}\approx \frac{\sigma_{0}E}{1+\mu^{2}B_{0}^{2}\cos^{2}\theta }$$

We can then deduce the magnetoresistance, for a *z*‐oriented magnetic field, as

(9) $$MR=\frac{R_{B}-R_{0}}{R_{0}}=\frac{j_{0}-j_{B,E}}{j_{B,E}}=\mu^{2}B_{0}^{2}\cos^{2}\theta$$

Despite the Hall potential that must form across the width of the domain wall in the conical geometry, we still develop the same kind of quadratic variation of magnetoresistance with magnetic field, as is seen in the Corbino disk. The correction factor of cos^2^θ is the same as one might expect from considering only the component of current in the cone which is influenced by magnetic field, i.e., by ignoring the *z*‐component of current and projecting the cone onto the plane perpendicular to the cone axes (visualized in Figure 2b,c).

**Figure 3:**
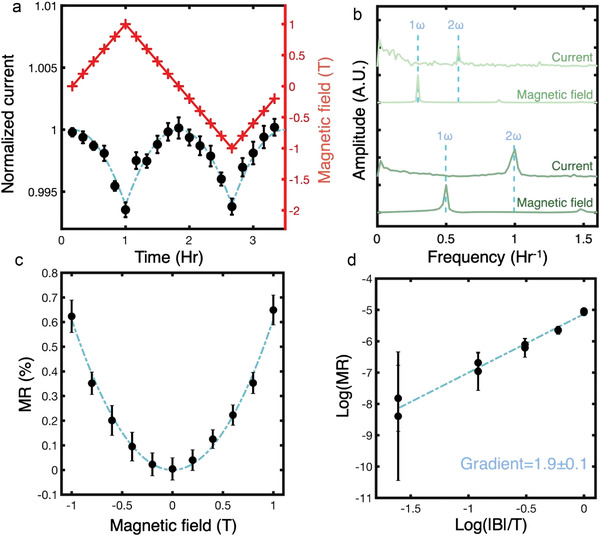
Geometric magnetoresistance (MR) measurement at conducting LNO domain walls. a) The MR response. The red “plus” motifs show the applied external magnetic field and the black circles show the normalized current response, averaged over 20 cycles. The blue dashed lines are parabolic fits (Equation (9)). Note that all fits are weighted by the uncertainty on each data point. b) Discrete fast Fourier transforms of the measured raw current and magnetic field profiles, shown for two datasets. c) A plot of the magnetic field versus the MR. The blue dashed line is a fitted parabola with *B* ^2^ coefficient (6.1 ± 0.5) × 10^−3^. d) A plot of log(*B*) versus log(MR), with a linear fit of coefficient 1.9 ± 0.1, illustrating the quadratic nature of the *B*–MR relationship, as expected from our geometric MR treatment.

Quantitative interpretations of geometric magnetoresistance are restricted by a low field limit, which dictates that *μB* is small (see Section S2 in the Supporting Information). Therefore, the expected resistance change might only be of the order of 1% or less, depending on the carrier mobility. To confidently isolate such a small signal, a periodic, linearly varying magnetic field profile was applied to the sample over several days, with current measurements taken continuously. The periodicity of the applied field (triangular waveform) allowed identification of the magnetoresistance signal in frequency space (**Figure** **3**b) as well as an averaging of the current response over many magnetic field cycles. The current response (averaged over 20 cycles) is plotted in Figure 3a along with the magnetic field profile used. A decrease in current with increasing magnetic field strength is clearly visible. Importantly, current varies with the magnitude of the applied magnetic field and is independent of its sign. Moreover, its form is parabolic (following Equation (9)). Results from two separate experiments with differing frequencies of applied magnetic field cycles are shown in Figure 3b. Clearly, the current response exhibits a principal component at the second harmonic frequency, as expected. Figure 3c shows the parabolic form of the magnetoresistance explicitly with a fitted *B* ^2^ coefficient of (6.1 ± 0.5) × 10^−3^. This was the largest magnetoresistance that was exhibited across all samples studied (presumably with the lowest contact resistance, as a large magnetically inactive in‐series contact resistance will depress the magnetoresistance (MR) signal). Figure 3d expresses the same information logarithmically, allowing direct extraction of the squared exponent in *B*.

To determine the carrier mobility, we apply Equation (9), assuming an angle of inclination θ of ≈78°. This gives a corrected room temperature mobility of μ ≈ 3700 cm^2^ V^−1^ s^−1^, an unusually high value for oxide semiconductors at room temperature. The estimated inclination angle is consistent with microstructural observations shown in Figure 1d, and with geometrical arguments associated with the domain diameter needed for significant source–drain currents to develop.^[^ ^19^ ^]^

**Figure 4:**
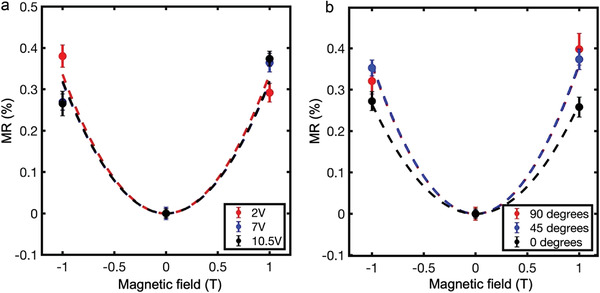
Further MR measurements. a) Magnetic field versus MR for LiNbO_3_ domain wall conduction, as a function of various applied voltages (and therefore as a function of current density). b) MR versus magnetic field for various orientations of the magnetic field. At 0°, the field is in the same orientation as illustrated in Figure 2, and at 90°, the field is in the *x–y* plane.

The magnetoresistance measurement was repeated on a separate sample, with different applied voltages (**Figure** **4**a) used to induce different current densities in the domain walls. No observable change in the magnetoresistance is seen and so it appears that the carrier mobility is insensitive to current density variations (at least, at these applied voltage levels which do not cause further ferroelectric switching).

The inferred mobility measurement allows for an estimation of carrier density, provided the current density is known. Other in‐house studies and published works^[^ ^19^ ^]^ have observed huge currents through LNO domain walls, of the order of 0.1 A for an array of domains similar to that shown in Figure 1. Taking cylindrical domain arrays with a radius of order *r* = 10^−7^ m and estimating a top electrode size of the order *A*_E_ ≈ 1 × 10^−9^ m^2^, with a driving field *E* = 10^7^ V m^−1^ (typical values used when driving ≈0.1 A through our two‐terminal multidomain wall structures), we can estimate the active carrier density (*n*_active_) as

(10) $$n_{\text{active}}=\frac{I}{A_{E}\frac{2\pi rd}{\pi r^{2}}E\mu e}\approx \frac{10^{13}}{d}\;m^{-3}=10^{9}\;{\text{cm}}^{-2}$$

where μ is the carrier mobility, *e* is the electronic charge, and *d* is the domain wall thickness, in meters. Note that this is a low‐end estimate, as any effects from parasitic contact resistance are not included in Equation (10). Contact resistance will act to reduce the measured MR signal and hence also reduce the inferred mobility. An estimate of the carrier density in two dimensions implied by full screening of the polar discontinuity at the wall is

(11) $$n_{\text{screen}}=\frac{2P\cdot S}{eS}\approx 10^{14}\;{\text{cm}}^{-2}$$

where *P* is the spontaneous polarization of LNO (≈0.7 C m^−2^) and again a domain wall inclination angle of 78° between the polarization and the domain wall surface normal is used. The ratio of *n*_active_ to *n*_screen_ (≈10^−5^) indicates that a relatively small fraction of the screening charge, assumed to be present, actually contributes to conduction, commensurate with previously obtained Hall effect measurements on domain walls in ErMnO_3_.^[^ ^32^ ^]^

## 4 Implications of the Magnetoresistance Observations

We observe a quadratic fractional increase in domain wall resistance with increasing magnetic field strength. While the quadratic response is characteristic of geometric magnetoresistance (as discussed and derived above), there are additional magnetotransport phenomena which could also be at play. In particular, the so‐called physical magnetoresistance is known to often have a quadratic dependence on magnetic field strength^[^ ^35^, ^56^, ^59^ ^]^ and could exist in our “Corbino cones,” in addition to the geometric effect. The challenge is then to disentangle magnetoresistance contributions, due to the absolute magnitude of mean carrier mobility (geometric), from those due to a spread in mobility values (physical). Prior studies^[^ ^35^, ^49^, ^56^, ^60^ ^]^ conclude that, when both effects are simultaneously present, geometric magnetoresistance dominates the overall behavior.

Furthermore, previous domain wall magnetotransport studies found either no magnetoresistance^[^ ^38^ ^]^ or an effect with a magnitude inconsistent with physical magnetoresistance,^[^ ^39^ ^]^ suggesting that significant spread in carrier mobility is not a general property of domain wall transport. In the minority of cases, in other material systems, where the carrier mobility spread is greater than the mean mobility, a nonsaturating linear magnetoresistance is expected^[^ ^61^ ^]^ and should persist into the low‐field regime. We see no hint of linearity in our measurements. Other domain wall studies, involving much larger fields,^[^ ^38^ ^]^ have seen no linear effects either, and this is commensurate with the view that carrier mobilities are much greater than the spread in carrier mobilities in conducting domain wall systems generally. Extraordinary MR can occur in specialized metal–semiconductor hybrid systems. Neither the nature of our system nor the magnitude of the observed MR is consistent with an extraordinary MR effect.

Figure 4b shows the effect of changing the angle between the applied magnetic field and the polar axis of the LNO films (the *z*‐axis in Figure 2b). The magnitude of the magnetoresistance measured in this instance was generally lower than shown in Figure 3. We attribute this to a larger contact resistance and inferior wire‐bond. Nevertheless, data clearly show that there is no significant variation in the magnetoresistance with magnetic field orientation. This surprising observation demands explanation. We have therefore derived an expression for the geometric magnetoresistance (again using an iterative approach), for magnetic fields applied parallel to the *x–y* plane (MR_*xy*_). The full treatment is given in Section S9 (Supporting Information). It is more complicated than that associated with the field oriented parallel to the polar axis, as Lorentz forces cause diametrically opposite strips of carrier accumulation and denudation to develop (Figure S9b, Supporting Information). This builds a Hall potential, such that the conical domain walls act as two “half‐cone” short Hall bars, connected electrically in parallel. Short Hall bars, with intermediate length‐to‐width ratios, allow geometric magnetoresistance to persist, albeit reduced by a prefactor dependent on aspect ratio. Using our derived form of the MR_*xy*_ (Section S9, Supporting Information), along with an analysis from Lippmann and Kuhrt (to estimate the aspect ratio prefactor),^[^ ^58^ ^]^ we find the resultant magnetoresistance (for the *x–y* plane oriented field) to be

(12) $${\text{MR}}_{xy}=\frac{0.25\mu^{2}B_{0}^{2}\sin^{2}\theta }{2}$$

Using this expression and assuming the geometric magnetoresistance, most accurately, represented in Figure 3, to be constant with respect to magnetic field orientation (as implied by Figure 4b) yields an alternative estimate of room‐temperature carrier mobility of ≈2000 cm^2^ V^−1^ s^−1^; this is lower than that given by Equation (9), but by less than a factor of 2 (reasonable given the coarse nature of some of the assumptions made in deriving Equation (12)).

Ultrahigh carrier mobility, accompanied by a low carrier density, should allow insight into the possible charge transport mechanisms at play. Defect‐related electron hopping seems unlikely (low carrier mobility is observed when hopping transport is dominant). Furthermore, defect aggregation at the domain walls is slow in LNO; defects seem largely insensitive to electric fields at room temperature (and so would not migrate to the charged wall to provide screening); this is evidenced by an extremely low bulk dark conductivity^[^ ^62^ ^]^ (10^−16^ to 10^−18^ Ω^−1^ cm^−1^). In any case, charged defect migration to domain walls has previously been blamed for a reduction, rather than increase, in conductivity.^[^ ^63^ ^]^

Fundamental alteration in the electronic band structure at the LNO domain wall may be possible. A reduction of the band gap, as has been seen in bismuth ferrite (BFO),^[^ ^30^ ^]^ would clearly lead to an increase in the electron population in the conduction band at room temperature. The ultrahigh carrier mobilities observed may also indicate a strong curvature developing at the bottom of the conduction band (which is not present in bulk LNO^[^ ^64^ ^]^) consistent with low effective carrier masses. Notable transport behavior has been seen at surfaces in conventional bulk semiconductors before, particularly at heterointerfaces. Like LNO, perovskite KTaO_3_ (KTO) displays an innocuous electronic band structure in bulk.^[^ ^65^, ^66^ ^]^ However, more detailed band structure measurements^[^ ^67^ ^]^ on the (111) face revealed a 2D electron gas (2DEG) with the potential for exotic transport properties. Superconductivity, for example, has been observed along heterointerfaces of (111) planes in KTO and EuO or LAO at low temperatures.^[^ ^68^ ^]^

Even metallic conduction can be considered. Current–voltage measurements as a function of temperature (Section S8, Supporting Information, and refs. [63, 69]) make this speculative, but the two‐probe measurement geometry suffers from series contact resistances, which may obscure any observation of metallicity. We discuss the theoretical possibility of a metal–insulator transition in Section S8 (Supporting Information), and note that further investigation of domain wall transport by four‐probe methods is required to give greater insight.

## 5 Summary and Outlook

We have performed novel geometric magnetoresistance measurements on conical conducting domain walls in lithium niobate. These measurements represent the first detailed magnetotransport study in this 2D system, and suggest a uniquely large room‐temperature mobility. Potential conduction mechanisms are discussed, but further and similar measurements at cryogenic temperatures are needed to fully elucidate the transport in these systems.

## 6 Experimental Section

### Sample Preparation

The sample consisted of a 500 nm‐thick *z*‐cut single crystal of lithium niobate, commercially available from NanoLN and prepared by the ion‐slicing method. It included a gold bottom electrode and a SiO_2_–LNO insulating substrate. For most of the current measurements, including the magnetoresistance measurements, silver thin‐film electrodes (≈20 nm thick) were deposited onto the surface of the LNO film through hardmask grids in 100 × 100 μm^2^ via thermal evaporation; the bottom electrode (already present in the as‐received samples) was contacted by coating the side with conducting silver electrodes. A 25 μm‐diameter aluminum wire was wire‐bonded to the silver top electrode by the application of ultrasonic pulses, providing robust electrical connections for the experiment. The as‐grown wafer was monodomain with its polarization pointing away from the bottom electrode. Conducting domain walls were bias‐written, with the sample connected in series to a 100 kΩ current‐limiting resistor. This both protects the electrode from high‐current‐density pulses during the switching process and produces a domain structure of sufficient resistance (≈ 5 MΩ), such that the current level was kept relatively low (around 1 μA at 5 V) throughout the MR experiment. A series of “writing” voltage pulses was applied to the series circuit from 0 to 50 V, and back down to 0 V in steps of 1 V, using a Keithley 237 source‐measure unit. After each pulse, a “reading” pulse of 5 V was applied to check the conducting state of the sample. It was found that a voltage pulse of roughly 26 V was enough to produce obvious domain wall conducting pathways through the sample.

### PFM/cAFM

PFM and topography measurements were carried out simultaneously with an MFP‐3D infinity AFM system from Asylum research. Conducting silver paste contacted the bottom electrode of the sample and was grounded, while an AC voltage was applied to a standard Pt‐coated Si AFM tip. The frequency of the AC voltage was tuned to match the resonant frequency of the tip–sample system (roughly 330 kHz) and the amplitude of the applied signal was varied between 1 and 4 V. For cAFM measurements, a steady DC bias of the order of −5 V was applied to the LNO bottom electrode, while the tip, in contact with the LNO surface, was held at ground. Current was then measured as a function of position on the sample surface.

### Magnetoresistance Measurements

For LNO domain walls, the sample was placed between the electromagnet pole pieces, where the magnetic induction might be varied in strength, up to a magnitude of 1 T, and monitored via an external Hall probe. In the standard measurement, current was driven through the conducting domain walls via the application of a constant 5 V bias to the surface silver electrode, using a Keithley 237 source‐measure unit, and measured as a function of magnetic field. The current was allowed through the sample to stabilize for 2 h to a noise level of roughly ±10 nA before applying the magnetic field profile, shown in Figure 3a. Given the low field limit mentioned above, the magnitude of the magnetoresistance signal was expected to be small, comparable to the noise level in the system. For this reason, 20 cycles of the *B* field profile were applied, as shown in Figure 3a, which consisted of 20 linearly spaced field steps between +1 and −1 T. Each field was applied for 600 s, bringing the total time for the experiment to ≈ 67 h. Repetition of the current measurements and magnetic field cycles allowed for improving the ±10 nA noise to produce the uncertainties in Figures 3 and 4. The error bars reflected the standard error on measurements of current across the multiple field cycles. Given the long timeline for the experiment, long‐scale current variations were also apparent. The current response of each magnetic field cycle was normalized with respect to the base current (*I* (*B* = 0)) for that cycle. Current measurements were made continuously at each field step, and the current value at any magnetic field was the average of 800 data points taken during that step. The average of corresponding current measurements across 20 magnetic field cycles produced the current data in Figure 3. Various current densities were investigated by applying larger voltages to a similar sample (Figure 4a). The field angle was investigated by rotating the sample mounting stub within the field.

### Control Experiment

A control test was also carried out. The magnetoresistance experiment was repeated, with current shorted through the bottom electrode of the same LNO sample used in the main experiment (Figure 3), effectively excluding the LNO film and domain walls from the circuit. No magnetoresistance was seen, eliminating the possibility that observations were due to spurious signal or experimental artifact. These control results are shown in Section S6 (Supporting Information).

### Current Component Calculation

The calculation of current components under electric and magnetic fields was carried out following a method described by Popovic.^[^ ^35^ ^]^ The rationale is as follows: carriers are initially assumed to move under the influence of the electric field only, giving a current density. Once the carriers drift, they experience the magnetic part of the Lorentz force, giving a second current density component, perpendicular to the first. This component is then deflected, and so on, and is calculated by *j*_*n*+1_ = *μ*(*j_n_* × *B*). Successive current “deflections” are summed, giving a final current density, which is accurate to higher powers of *μB*, depending on the degree of iteration. How this method provides a solution which converges toward the analytic solution for current density in the Corbino disk is shown in the Supporting Information.

To solve for the current in the Corbino cone, insulating boundary conditions were further considered: any current component that arises, which is orientated toward an insulating boundary, is cancelled by an equal and opposite “Hall” component. Using this method, the equation for MR was arrived at the conical conducting domain walls.

### Transmission Electron Microscopy

Cross‐sectional transmission electron microscopy (TEM) samples were prepared by ion milling, using a dual‐beam focused ion beam (FIB) microscope. When thinned to electron transparency, the sample was mounted onto an Omniprobe copper lift‐out grid. The scanning TEM (STEM) imaging was performed using a Thermo‐Fisher Scientific double tilt TEM holder in the Thermo‐Fisher Scientific FEI double aberration‐corrected Titan Themis Z, located at the University of Limerick, operated at 300 kV.

## Conflict of Interest

The authors declare no conflict of interest.

## Authors Contribution

The magnetoresistance experimental design, data collection, and data analyses were carried out by C.J.McC. and M.G.C. Electrical characterization with temperature was performed by C.J.McC. The details of the MR model were derived by C. J. McC. and J.P.V.McC. PFM and cAFM were carried out by J.R.M. and J.P.V.McC. STEM measurements, elucidating the domain wall microstructure, were carried out by M.C., K.M., A.H., U.B., and J.P.V.McC. The experimental idea was conceived by J.P.V.McC. and J.M.G. J.M.G supervised the project along with R.G.P.McQ. and A.K. M.B. contributed expertise in magnetoresistance measurement and interpretation. All authors contributed to the discussion and interpretation of results, and all were involved in the manuscript preparation.

## Supporting information

Supporting Information

## Data Availability

The data that support the findings of this study are available from the corresponding author upon reasonable request.
