# Supplementary material for: Ultrahigh Carrier Mobilities in Ferroelectric Domain Wall Corbino Cones at Room Temperature
Source: Adv Mater. 2022 Jul 11;34(32):2204298. doi: 10.1002/adma.202204298 (PMC11475267; doi:10.1002/adma.202204298)
Supplement: Supplementary file 1 — Supporting Information [file ADMA-34-2204298-s001.pdf]

ADVANCED MATERIALS

Supporting Information

for *Adv. Mater.,* DOI: 10.1002/adma.202204298

Ultrahigh Carrier Mobilities in Ferroelectric Domain Wall Corbino Cones at Room Temperature

Conor J. McCluskey, Matthew G. Colbear, James P. V. McConville, Shane J. McCartan, Jesi R. Maguire, Michele Conroy, Kalani Moore, Alan Harvey, Felix Trier, Ursel Bangert, Alexei Gruverman, Manuel Bibes, Amit Kumar, Raymond G. P. McQuaid, and J. Marty Gregg**

Ultrahigh Carrier Mobilities in Ferroelectric Domain Wall Corbino Cones at Room Temperature

Supplementary information

Conor J. McCluskey, Matthew G. Colbear, James P. V. McConville, Shane J. McCartan, Jesi R. Maguire, Michele Conroy, Kalani Moore, Alan Harvey, Felix Trier, Ursel Bangert, Alexei Gruverman, Manuel Bibes, Amit Kumar, Raymond G. P. McQuaid and J. Marty Gregg

Section S1: Derivation of current density in a material in the presence of E and B fields

The following derivation follows Popovic[1]. In relatively low fields, we approximate the continuous acceleration and scattering of the charge carrier (of charge $-q$ and effective mass m^*) as a smooth drift, with drift velocity $\boldsymbol{v_d} = -\mu \boldsymbol{E_0}$, where μ is the carrier mobility and $\boldsymbol{E_0}$ is the external electric field. Writing the Lorentz force due to external electric and magnetic fields ($\boldsymbol{E_0}$ and $\boldsymbol{B}$) as an effective electric field $\boldsymbol{E_{eff}}$, we state:

$$-q\boldsymbol{E_{eff}} = -q\boldsymbol{E_0} - q(\boldsymbol{v_d} \times \boldsymbol{B}) \quad \text{S 1}$$

Note here that the magnetisation in non-magnetic $LiNbO_3$ (LNO) is zero, such that an external magnetic field $\boldsymbol{H}$ and magnetic induction $\boldsymbol{B}$ are proportional. Writing the Lorentz force as an equivalent electrical force allows us to readily represent the resultant current density in the material, in terms of external fields. By multiplying by $-\mu n$, we can write:

$$\boldsymbol{j_B} = \boldsymbol{j_0} - \mu(\boldsymbol{j_B} \times \boldsymbol{B}) \quad \text{S 2}$$

where μ is the carrier mobility, and n the carrier density. $\boldsymbol{j_B}$ and $\boldsymbol{j_0}$ are the current densities in the presence and absence of magnetic field, respectively. The dot product of $\boldsymbol{j_B}$ with $\boldsymbol{B}$ is

$$\boldsymbol{j_B} \cdot \boldsymbol{B} = \boldsymbol{j_0} \cdot \boldsymbol{B}. \quad \text{S 3}$$

while the cross product of $\boldsymbol{j_B}$ with $\boldsymbol{B}$ is:

$$\boldsymbol{j_B} \times \boldsymbol{B} = \boldsymbol{j_0} \times \boldsymbol{B} - \mu[(\boldsymbol{j_B} \times \boldsymbol{B}) \times \boldsymbol{B}] \quad \text{S 4}$$

Using the vector identity:

$$(\boldsymbol{A} \times \boldsymbol{B}) \times \boldsymbol{C} = \boldsymbol{B}(\boldsymbol{A} \cdot \boldsymbol{C}) - \boldsymbol{A}(\boldsymbol{B} \cdot \boldsymbol{C}) \quad \text{S 5}$$

and equation S 4, we can write:

$$\boldsymbol{j_B} \times \boldsymbol{B} = \boldsymbol{j_0} \times \boldsymbol{B} - \mu[\boldsymbol{B}(\boldsymbol{j_B} \cdot \boldsymbol{B}) - \boldsymbol{j_B}(\boldsymbol{B} \cdot \boldsymbol{B})] \quad \text{S 6}$$

After replacing $\boldsymbol{j_B} \cdot \boldsymbol{B}$ using equation S 3, S 5 becomes

$$\boldsymbol{j_B} \times \boldsymbol{B} = [\boldsymbol{j_0} \times \boldsymbol{B}] - \mu[\boldsymbol{B}(\boldsymbol{j_0} \cdot \boldsymbol{B}) - \boldsymbol{j_B}(\boldsymbol{B} \cdot \boldsymbol{B})] \quad \text{S 7}$$

Substituting S 7 into S 2 we arrive at:

$$\boldsymbol{j_B} = \frac{\boldsymbol{j_0} - \mu(\boldsymbol{j_0} \times \boldsymbol{B}) + \mu^2 \boldsymbol{B}(\boldsymbol{j_0} \cdot \boldsymbol{B})}{\boldsymbol{1} + \mu^2 B^2} \quad \text{S 8}$$

In terms of the external fields:

$$\boldsymbol{j_B} = \frac{\sigma_0 \boldsymbol{E_0} + \sigma_0 \mu\, (\boldsymbol{E_0} \times \boldsymbol{B}) + \mu^2 \sigma_0 \boldsymbol{B}(\boldsymbol{E_0} \cdot \boldsymbol{B})}{1 + \mu^2 \boldsymbol{B}^2} \quad \text{S 9}$$

if we change μ such that it carries the sign of carriers. Here, σ_0 is the conductivity of the material when $\boldsymbol{B} = 0$. This is the result quoted in the main text. Note that, if we include a Hall field contribution, we can replace $\boldsymbol{E_0}$, in equation S 9, with $\boldsymbol{E_0} + \boldsymbol{E_H}$, where $\boldsymbol{E_H}$ is the Hall field in the system.

Section S2: The low-field condition

To recover the typical magnetoresistance (MR) response[1]:

$$MR = \mu^2 B^2 \qquad \text{S 10}$$

a smooth drift approximation and low field condition are needed. We now quantify this condition by realising that the applicability of the smooth drift approximation is related to the fraction of a full cyclotron orbit that a carrier can complete between any two scattering events. If the mean free collision time $\langle\tau\rangle$ is much smaller than the time for a cyclotron orbit (T_C), the drift is correctly identified as smooth, and regular collisions represent a smooth frictional type force. Physically, as the particle begins its cyclotron orbit under the crossed $\boldsymbol{B}$ and $\boldsymbol{E}$ fields, it collides with the lattice, loses its energy and starts again before completing a significant portion of the orbit. This process repeats, and the resultant is approximately a straight-line path, inclined with respect to the external electric field, with a magnetoresistance response that follows S 10. If several cyclotron orbits are completed between two collisions, the effect of the magnetic field on the carrier is clearly more severe, and our smooth drift breaks down[1]. In the high field case, the MR behaviour therefore departs from S 10. Given the cyclotron orbital period:

$$T_C = \frac{2\pi m^*}{qB} \qquad \text{S 11}$$

and the definition of carrier mobility μ in terms of mean free transit time between scattering collisions $\langle\tau\rangle$

$$\mu = \frac{q\langle\tau\rangle}{m^*} \qquad \text{S 12}$$

the “low field condition”, defined by $\langle\tau\rangle \ll T_c$, becomes:

$$\mu B \ll 2\pi \qquad \text{S 13}$$

Section S3: MR and Hall electric field in the Corbino disc

The main consideration in the extension from the Corbino disc to a cone geometry is that the $\boldsymbol{E_0} \cdot \boldsymbol{B}$ term can be zero in the disc (for the typical case of $\boldsymbol{E} \vdash \boldsymbol{B}$ **),** whereas for current confined to the cone, this is necessarily not true. The cone is differentiated from a disc by a *z* height, through which current must pass to traverse along the walls, with *z* also being the axis parallel to the magnetic field. Therefore, there is a component of current along the magnetic field direction. The analytic result of equation S 9 is general, representing the current density in a material for arbitrarily aligned $\boldsymbol{E}$ and $\boldsymbol{B}$ fields and in the absence of Hall potential build up. Here, we consider the effect of this general solution in disc-like and conical geometries explicitly, showing the similarities and differences.

Beginning with the simpler case of the disc, set up such that $\boldsymbol{E} \perp \boldsymbol{B}$, we use cylindrical coordinates to reflect the symmetry of the system. Here, the driving electric field is radial and the magnetic field is along z:

$$\boldsymbol{E_0} = \begin{pmatrix} E_r \\ E_\varphi \\ E_z \end{pmatrix} = E_0 \begin{pmatrix} 1 \\ 0 \\ 0 \end{pmatrix} \qquad \text{S14}$$

$$\boldsymbol{B} = \begin{pmatrix} B_r \\ B_\varphi \\ B_z \end{pmatrix} = B_0 \begin{pmatrix} 0 \\ 0 \\ 1 \end{pmatrix} \qquad \text{S15}$$

where r, φ and z denote the usual radial, azimuthal and vertical unit vectors of cylindrical coordinates. E_0 and B_0 are electric and magnetic field amplitudes. Using the analytic result from S 9, we find a current density of

$$\boldsymbol{j_{disc}} = \frac{\sigma_0 E_0}{1 + \mu^2 {B_0}^2} \begin{pmatrix} 1 \\ -\mu B_0 \\ 0 \end{pmatrix} \qquad \text{S16}$$

This is the classic result obtained for the magnetoresistance in a Corbino disc. The current density along $\hat{\boldsymbol{r}}$ (the external electric field direction, giving the device current) is reduced by a factor $\frac{1}{1+\mu^2 {B_0}^2}$, allowing for the appearance of an azimuthal component along $\hat{\boldsymbol{\varphi}}$ which is dependent on the magnetic field strength. As mentioned, no Hall potential builds in the case of the Corbino disc with a surface-normal magnetic field. This means that the solution in S16 is exact.

Section S4: An iterative approach for calculating current density in the Corbino disc

It is useful to analyse this same problem using an iterative step like approach[1], as applied by Popovic, because this is the method which can be used to analyse situations beyond the Corbino disc, where Hall potentials can form. In this iterative method, an initial estimate for current is given as the usual drift due to the electric field. Then, Lorentz deflection of each current component that appears is calculated, and progressively smaller corrections to the current density are made. Given that the full solution is accurate for the disc, we here use the iterative solution and compare it to the analytic solution S16.

Starting with $\boldsymbol{j_0}$ as the ordinary radial current component due to the electric field:

$$\boldsymbol{j_0} = \sigma_0 \boldsymbol{E_0} = \sigma_0 E_0 \begin{pmatrix} 1 \\ 0 \\ 0 \end{pmatrix} \qquad \text{S 17}$$

the Lorentz deflection of this current component produces $\boldsymbol{j_1}$, the azimuthal component, which is orientated along $\boldsymbol{j_0} \times \boldsymbol{B}$. Here we write $\boldsymbol{j_1}$ in terms of an effective electric field $\boldsymbol{E_1}$, which can be interpreted as the electric field that would produce the same force on the particle as the magnetic part of the Lorentz force acting on the carrier:

$$q\boldsymbol{E_{eff}} = q(\boldsymbol{v_d} \times \boldsymbol{B}) \qquad \text{S 18}$$

Then we can rewrite $\boldsymbol{j_1}$ in terms of the externally applied fields:

$$\boldsymbol{j_1} = \sigma_0 \boldsymbol{E_1} = \sigma_0(\boldsymbol{v_{d0}} \times \boldsymbol{B}) = \sigma_0 \mu\, (\boldsymbol{E_0} \times \boldsymbol{B}) = \mu(\boldsymbol{j_0} \times \boldsymbol{B}) \qquad \text{S 19}$$

Here, $\boldsymbol{v_{d0}}$ is the drift velocity of the original current component $\boldsymbol{j_0}$, upon which the Lorentz force acts.

Evaluating $\boldsymbol{j_1}$ gives:

$$\boldsymbol{j_1} = \mu(\boldsymbol{j_0} \times \boldsymbol{B}) = \sigma_0 E_0 \begin{pmatrix} 0 \\ -\mu B_0 \\ 0 \end{pmatrix} \qquad \text{S 20}$$

Subsequent terms can be determined in the same way as in S 19. The next term is the Lorentz deflection of the azimuthal component $\boldsymbol{j_1}$, and produces a third component, $\boldsymbol{j_2}$, opposing the original motion of the carrier:

$$\boldsymbol{j_2} = \mu(\boldsymbol{j_1} \times \boldsymbol{B}) = \sigma_0 E_0 \begin{pmatrix} -\mu^2 {B_0}^2 \\ 0 \\ 0 \end{pmatrix} \qquad \text{S 21}$$

This leads to a reduction in current density along $\boldsymbol{E_0}$, and gives the first approximation of the geometric magnetoresistance. The next term $\boldsymbol{j_3}$ adds a correction to the azimuthal current:

$$\boldsymbol{j_3} = \mu(\boldsymbol{j_2} \times \boldsymbol{B}) = \sigma_0 E_0 \begin{pmatrix} 0 \\ \mu^3 {B_0}^3 \\ 0 \end{pmatrix} \qquad \text{S 22}$$

The process can be continued to develop further higher order corrections to the current.

If we continue the cycle, we obtain a solution of the form

$$\boldsymbol{j_{disc}} = \sigma_0 E_0 \begin{pmatrix} (1 - \mu^2 {B_0}^2 + \mu^4 {B_0}^4 - \mu^6 {B_0}^6 \dots) \\ -\mu B_0 (1 - \mu^2 {B_0}^2 + \mu^4 {B_0}^4 - \dots) \\ 0 \end{pmatrix} \qquad \text{S 23}$$

Where we can make use of the expansion around $x = 0$:

$$\frac{1}{(1+x)} = 1 - x + x^2 - x^3 \dots \qquad \text{S 24}$$

with $x = \mu^2 {B_0}^2$ to retrieve the full solution:

$$\boldsymbol{j_{disc}} = \frac{\sigma_0 E_0}{(1 + \mu^2 {B_0}^2)} \begin{pmatrix} 1 \\ -\mu B_0 \\ 0 \end{pmatrix} \qquad \text{S 25}$$

As an illustration to help visualise the approach, the first 3 components of the iterative solution are shown below in fig. S1.

The advantage of this iterative method is in detecting unexpected Hall field components. Any appearing current component that takes the carrier off the conducting surface and onto a sample boundary or insulating part of the sample can be accounted for immediately by including a mitigating Hall potential. That component and its subsequent Lorentz deflection then do not appear in calculations of further components. The full solution assumes the carrier is free to move in any direction, inaccurately predicting the current density when a Hall potential needs to be considered. A correct Hall field, if it is known, can be included in the analytic solution to give the correct current density (by simply replacing $\boldsymbol{E_0}$ in S 9 with $(\boldsymbol{E_0} + \boldsymbol{E_{Hall}})$). However, as of yet, we have not found a way to accurately assess the Hall field from the analytic solution alone. While the Corbino disc requires no Hall field correction, the case of the cone does, as is discussed below.

It is worth noting that while the methodology of the iterative solution appears to suggest that subsequent Lorentz deflections are occurring in time on a particle, this is not the physical picture. We are calculating, to higher order accuracy, the components of the effect of the magnetic field on a particle, directed along the major axes in the system.

a

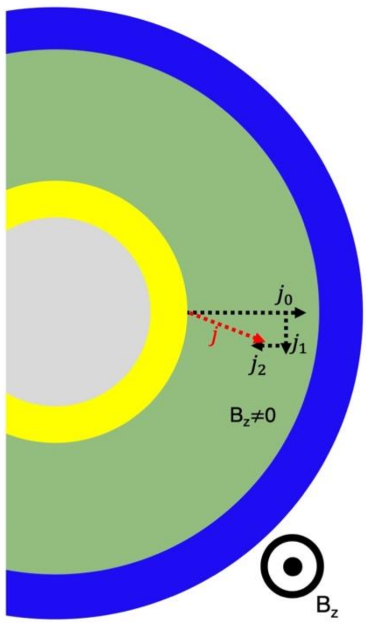

Figure S1| Iterative solution in the Corbino disc. a, Vectors of current density produced in the approximate description given above, for a current carrying Corbino disc in the presence of a magnetic field perpendicular to the disc surface.

Section S5: MR and Hall electric field in the cone

The main difference between the disc and cone is that, in order to drive current along the inclined cone, we now must have a z component of the applied electric field. Given that the z direction is the applied magnetic field direction, we now must have a component of current along the magnetic field direction, and the $\boldsymbol{E} \cdot \boldsymbol{B}$ term in S 9 no longer disappears. As we show below, if we assume that carriers, subjected to a conical electric field profile, are free to move in any direction (by using the analytic solution with no Hall field included), we find that the resultant current density will take carriers off the original conical surface.

Driving current along a general cone, we have electric and magnetic fields:

$$\boldsymbol{E_0} = \begin{pmatrix} E_r \\ E_\varphi \\ E_z \end{pmatrix} = E_0 \begin{pmatrix} -cos\,\theta \\ 0 \\ -sin\,\theta \end{pmatrix} \qquad \text{S 26}$$

$$\boldsymbol{B} = \begin{pmatrix} B_r \\ B_\varphi \\ B_z \end{pmatrix} = B_0 \begin{pmatrix} 0 \\ 0 \\ -1 \end{pmatrix} \qquad \text{S 27}$$

The inclination angle is α, defined as

$$tan\,\alpha = \left|\frac{E_z}{E_r}\right| = \frac{sin\,\theta}{cos\,\theta} = tan\,\theta \qquad \text{S 28}$$

$$\therefore \alpha = \theta \qquad \text{S 29}$$

Applying the analytic solution:

$$\boldsymbol{j_{cone}} = \frac{\sigma_0 E_0}{1+\mu^2 {B_0}^2}\begin{pmatrix} -\cos\theta \\ -\mu B_0 \cos\theta \\ 0 \end{pmatrix} + \sigma_0 E_0 \begin{pmatrix} 0 \\ 0 \\ -\sin\theta \end{pmatrix} \qquad \text{S 30}$$

Immediately, we notice that the radial component of the current density is reduced by a factor of $\frac{1}{1+\mu^2 {B_0}^2}$, making way for an azimuthal component which is dependent on the magnetic field. In this way the disc and cone are the same. The deviation between disc and cone can be seen when the *z* component of the current density, which remains unaffected by the magnetic field, is included. Its inclusion pushes carriers off the cone surface, as the ratio of $\boldsymbol{j_r}$ to $\boldsymbol{j_z}$ has changed. This effectively changes the inclination of the cone that would be swept out by carriers if they were free to move in any locus.

$$\tan\alpha = \left|\frac{E_z}{E_r}\right| = \frac{(1+\mu^2 {B_0}^2)\sin\theta}{\cos\theta} = (1+\mu^2 {B_0}^2)\tan\theta \qquad \text{S 31}$$

Of course, the conducting pathway is actually confined to the conical domain wall which, as far as we know, doesn't change shape during the magnetoresistance measurement. Given that lithium niobate is highly insulating, carrier deviation from the conducting cone will instead be counteracted by the formation of a Hall potential, which builds up across the conical domain wall width.

Therefore, we use an iterative solution to solve for the current density, allowing us to assess both Hall and magnetoresistive components which arise. $\boldsymbol{j_0}$**,** the component of conventional drift due to the electric field, now acts along the inclined wall of the cone:

$$\boldsymbol{j_0} = \sigma_0 E_0 \begin{pmatrix} -\cos\theta \\ 0 \\ -\sin\theta \end{pmatrix} \qquad \text{S 32}$$

The Lorentz deflection of $\boldsymbol{j_0}$ produces $\boldsymbol{j_1}$, acting in the direction of $\boldsymbol{E_0} \times \boldsymbol{B}$**.** Thus, $\boldsymbol{j_1}$ acts in the azimuthal direction, which is tangential to the cone surface. $\boldsymbol{j_1}$ is illustrated in Fig. S2a-b.

$$\boldsymbol{j_1} = \sigma_0 E_0 \begin{pmatrix} 0 \\ -\mu B_0 \cos\theta \\ 0 \end{pmatrix} \qquad \text{S 33}$$

This initial deflection would keep the carrier on the cone, as at all points $\boldsymbol{E_0} \times \boldsymbol{B}$ locally follows the surface of the cone, and this case is identical to the disc. Thus, we need not consider a Hall potential yet. The Lorentz deflection of $\boldsymbol{j_1}$ produces a force along $\boldsymbol{j_1} \times \boldsymbol{B}$**,** creating a $\boldsymbol{j_2}$. In the cone scenario, $\boldsymbol{j_1} \times \boldsymbol{B}$ is orientated **radially outwards** from the conical axis (note that this is not perpendicular to the cone surface), as shown in Fig. S2a-b. This component $\boldsymbol{j_2}$ is clearly not embedded in the cone, and so would result in a Hall potential.

$$\boldsymbol{j_2} = \sigma_0 E_0 \begin{pmatrix} \mu^2 {B_0}^2 \cos\theta \\ 0 \\ 0 \end{pmatrix} \qquad \text{S 34}$$

To assess the Hall electric field that this creates, we can split $\boldsymbol{j_2}$ up. The first component, $\boldsymbol{j_{2E}}$, is orientated against the original flow of current, reducing the current in the electric field direction. The same thing happens in the disc, and in both cases, this is the first approximation to the magnetoresistance. This component is given by:

$$\boldsymbol{j_{2E}} = \frac{\boldsymbol{j_2}\cdot\widehat{\boldsymbol{E}}}{\left|\widehat{\boldsymbol{E_0}}\right|^2}\widehat{\boldsymbol{E_0}} = \sigma_0 E_0 \begin{pmatrix} \mu^2 {B_0}^2 \cos^3\theta \\ 0 \\ \mu^2 {B_0}^2 \sin\theta\cos^2\theta \end{pmatrix} = -\mu^2 {B_0}^2 \cos^2\theta\, \boldsymbol{E_0} \qquad \text{S 35}$$

Since it is free to propagate, this component forms the basis of the next iteration. So $\boldsymbol{j_3}$ arises as the deflection of $\boldsymbol{j_{2E}}$, and so on. The other component of $\boldsymbol{j_2}$ we consider is parallel to the surface normal of the cone, $\widehat{\boldsymbol{u}}$. We call this component, $\boldsymbol{j_{2Norm}}$. This is illustrated in Figs S2a-b. Figs S2c-d show the components from the analytic solution for comparison. $\widehat{\boldsymbol{u}}$ is defined as the cross-product of the unit vector in the electric field direction, $\widehat{\boldsymbol{E}}$, and the unit vector in the azimuthal direction, $\widehat{\boldsymbol{\varphi}}$;

$$\widehat{\boldsymbol{u}} = \widehat{\boldsymbol{E}} \times \widehat{\boldsymbol{\varphi}} = \begin{pmatrix} \sin\theta \\ 0 \\ -\cos\theta \end{pmatrix} \qquad \text{S 36}$$

We can then calculate the Hall electric field which is needed to oppose and cancel this current component:

$$\boldsymbol{E_{2,Hall}} = \frac{-\boldsymbol{j_{2Norm}}}{\sigma_0} = -\frac{\boldsymbol{j_2}\cdot\widehat{\boldsymbol{u}}}{\sigma_0|\widehat{\boldsymbol{u}}|^2}\widehat{\boldsymbol{u}} = \mu^2 {B_0}^2 E_0 \cos\theta\sin\theta \begin{pmatrix} -\sin\theta \\ 0 \\ \cos\theta \end{pmatrix} \qquad \text{S 37}$$

The next iteration uses only equation S 35:

$$\boldsymbol{j_3} = \mu(\boldsymbol{j_{2E}} \times \boldsymbol{B}) = \sigma_0 E_0 \begin{pmatrix} 0 \\ \mu^3 {B_0}^3 \cos^3\theta \\ 0 \end{pmatrix} \qquad \text{S 38}$$

$\boldsymbol{j_3}$ is azimuthal and isn't altered by a Hall potential. Next is $\boldsymbol{j_4}$:

$$\boldsymbol{j}_4 = \mu(\boldsymbol{j_3} \times \boldsymbol{B}) = \sigma_0 E_0 \begin{pmatrix} -\mu^4 {B_0}^4 \cos^3\theta \\ 0 \\ 0 \end{pmatrix} \qquad \text{S 39}$$

We see a pattern emerge that every second calculated current component requires projection back onto the cone. The Hall field component of $\boldsymbol{j}_4$ is

$$\boldsymbol{E_{4,Hall}} = \frac{\boldsymbol{j_2}\cdot\widehat{\boldsymbol{u}}}{\sigma_0|\widehat{\boldsymbol{u}}|^2}\widehat{\boldsymbol{u}} = \sigma_0 E_0 \begin{pmatrix} \mu^4 {B_0}^4 \cos^3\theta\sin^2\theta \\ 0 \\ -\mu^4 {B_0}^4 \cos^4\theta\sin\theta \end{pmatrix} = \sigma_0 E_0\, \mu^4 {B_0}^4 \cos^3\theta\sin\theta\, \widehat{\boldsymbol{u}} \qquad \text{S 40}$$

And the electric field component

$$j_{4,E} = \frac{\boldsymbol{j_4}\cdot\widehat{\boldsymbol{E_0}}}{\left|\widehat{\boldsymbol{E_0}}\right|^2}\widehat{\boldsymbol{E_0}} = \sigma_0 E_0 \begin{pmatrix} -\mu^4 {B_0}^4 \cos^5\theta \\ 0 \\ -\mu^4 {B_0}^4 \cos^4\theta\sin\theta \end{pmatrix} = \sigma_0 E_0\, \mu^4 {B_0}^4 \cos^4\theta\, \boldsymbol{E} \qquad \text{S 41}$$

Summing the Hall components suggests a full solution for the Hall electric field:

$$\boldsymbol{E}_{Hall} = \boldsymbol{E_{2,Hall}} + \boldsymbol{E_{4,Hall}} \ldots = -\mu^2 {B_0}^2 E_0 \cos\theta\sin\theta(1 - \mu^2 {B_0}^2 \cos^2\theta + \mu^4 {B_0}^4 \cos^4\theta \ldots)\,\widehat{\boldsymbol{u}} \qquad \text{S 42}$$

Making use again of the series expansion in S 24:

$$E_{Hall} = -\frac{\mu^2 {B_0}^2 E_0 \cos\theta \sin\theta}{1 + \mu^2 {B_0}^2 \cos^2\theta}\,\hat{\mathbf{u}} \qquad \text{S 43}$$

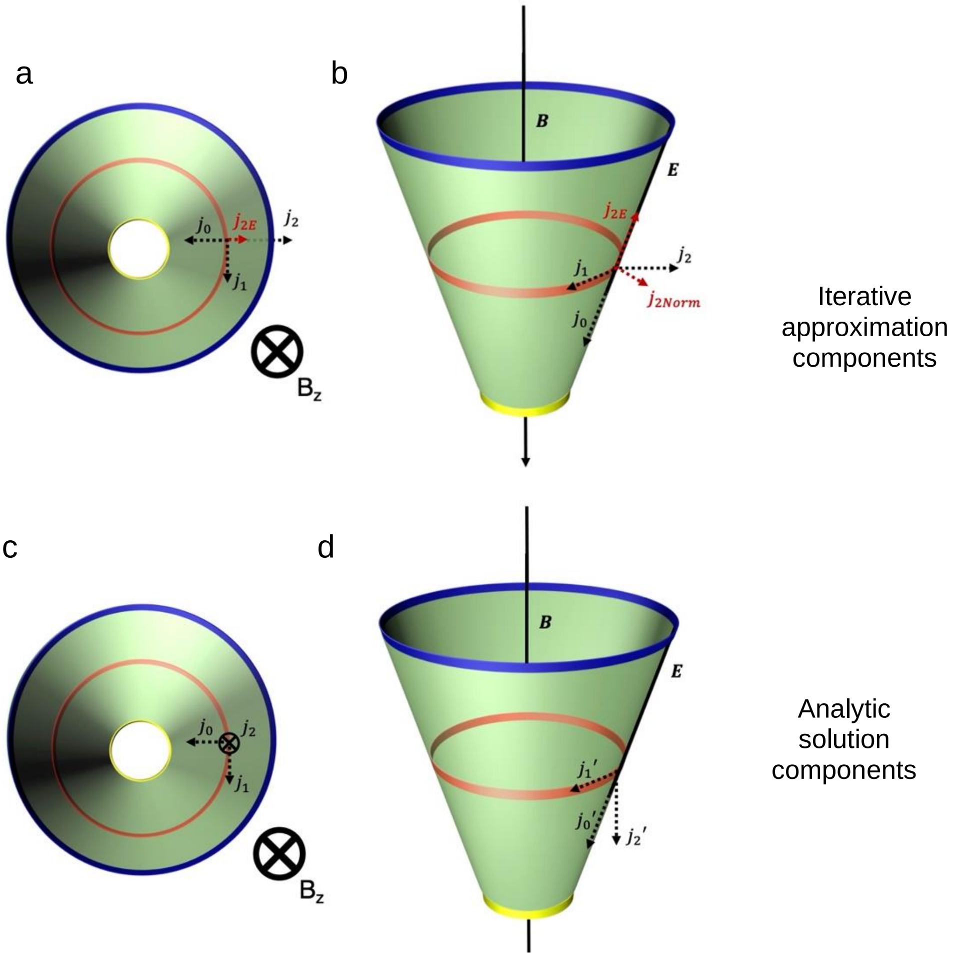

Figure S2| The Corbino cone. Plan view (a,c) and oblique view (b,d) of the LNO conducting wall, with the relevant current components as obtained from an iterative procedure (a,b) and analytically (c,d), which converge to the same result.

Key to this argument is the idea that only current components which lie in the cone itself can be allowed to propagate and become deflected. Any other components are eliminated by a Hall field component, as soon as they appear, such that their deflection isn't considered in future iterations. For example, only $\boldsymbol{j_{2E}}$ is used in the calculation of $\boldsymbol{j_3}$, rather than the full component $\boldsymbol{j_2}$.

Finally, we can sum the allowed components to produce the final current density in the cone:

$$\boldsymbol{j}_{total} = \boldsymbol{j_0} + \boldsymbol{j_1} + \boldsymbol{j}_{2,\boldsymbol{E}} + \boldsymbol{j}_3 + \boldsymbol{j}_{4,\boldsymbol{E}} \ldots \qquad \text{S 44}$$

$$= \sigma_0\left(1 - \mu^2 {B_0}^2 \cos^2\theta + \mu^4 {B_0}^4 \cos^4\theta \ldots\right)[\boldsymbol{E_0} + \begin{pmatrix} 0 \\ \mu B_0 \cos\theta \\ 0 \end{pmatrix}]$$

Which approximates a full solution of:

$$\boldsymbol{j_{total}} = \left[\frac{1}{1 + \mu^2 {B_0}^2 cos^2\theta}\right]\sigma_0 \boldsymbol{E_0} + \frac{\sigma_0 E_0}{1 + \mu^2 {B_0}^2 cos^2\theta}\begin{pmatrix} 0 \\ \mu B_0 \cos\theta \\ 0 \end{pmatrix} \qquad \text{S 45}$$

Where again the series expansion is used. The magnetoresistance can then be represented as the ratio between the magnitudes of the current components along the external electric field, in the presence and absence of the magnetic field:

$$\frac{j_E(0)}{j_E(B)} = \frac{\sigma_0 E_0}{\frac{1}{1+\mu^2 {B_0}^2 cos^2\theta}\sigma_0 E_0} = 1 + \mu^2 {B_0}^2 cos^2\theta \qquad \text{S 46}$$

giving a magnetoresistance

$$MR = \frac{j_E(0)}{j_E(B)} - 1 = \mu^2 {B_0}^2 cos^2\theta \qquad \text{S 47}$$

as quoted in the main text.

Section S6: Short test control experiment

As mentioned in the main text, we completed a control experiment, which tests the external circuit for spurious magnetoresistance. This was done to rule out the possibility that the magnetoresistance we assign to the lithium niobate domain walls is actually an artefact from the external circuit, or indeed arising from the AuCr bottom electrode, which could in principle demonstrate a magnetoresistance. To do this, the MR experiment was repeated with the same sample, save that the LNO film was excluded from the circuit by shorting across to the bottom AuCr electrode. In this way, all other circuit elements can remain the same, with only the LNO film and the connection to the film excluded. We present the results of this short test experiment below in Fig. S3, alongside the equivalent data for the conducting domain walls (Fig. 3c in the main text).

To keep the current consistent between experiments, a series resistor was added in the

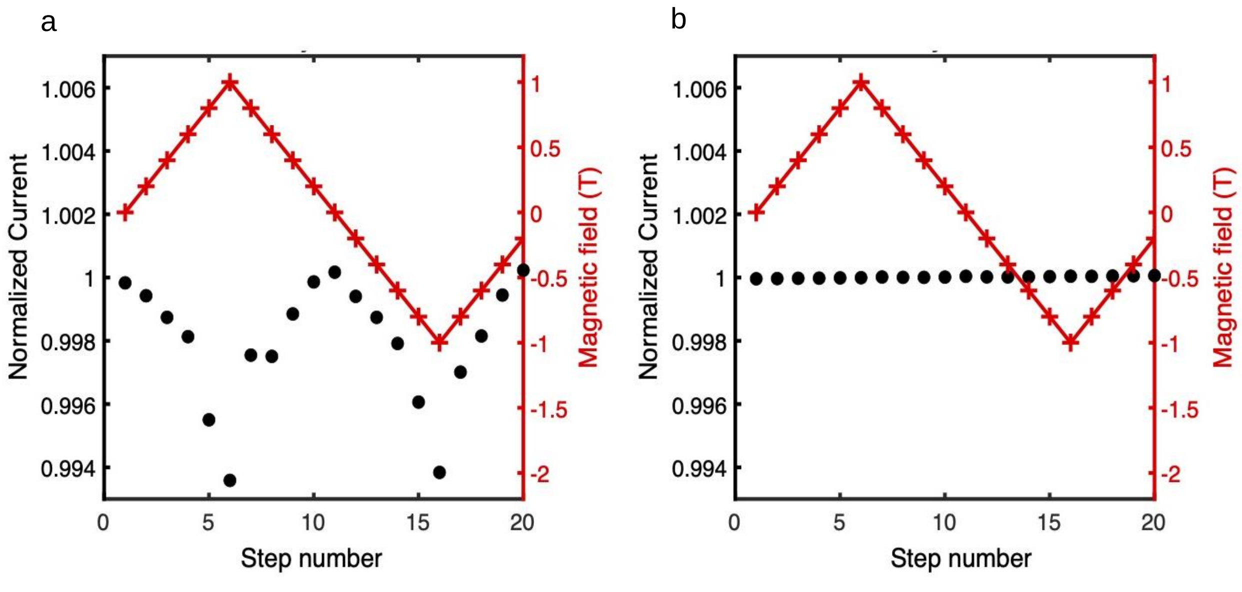

Figure S3 | Magnetoresistance control experiment. a, The magnetoresistance response of LNO domain walls, as shown in the main text. The red + show the applied external magnetic field, and the black circles show the normalised current response, averaged over 20 cycles. **b,** The magnetoresistance response during the control experiment. Here, the averaging is done over 13 cycles

control experiment, which replicates the resistance of the LNO film in the normal MR experiment. If the LNO is a bystander and the MR signal appears due to some other circuit element in our setup, then we should see an MR signal in this control experiment. Clearly, however, no variation of current with magnetic field exists, and we can hence rule out the notion that the MR is an external artefact.

Section S7: Magnetic field influence on domain structure

To ensure the magnetic field has no effect on the domain structure, we performed PFM mapping in a magnetic field. First, conducting domain walls were injected into the as-received LNO, using an AFM tip as the top electrode. PFM domain mapping was carried out before and after magnetic field cycling, which was done using the same magnet as in the MR measurements (Fig. S4). No changes in domain structure were seen.

Then, PFM was performed while a magnetic field was applied to the sample in situ (Fig. S5). The AFM magnetic field unit is designed to apply an in-plane magnetic field to the system (Fig. S5a). No changes to the domain structure were seen with the field in this in-plane geometry (Fig. S5b-d). We accessed the out-of-plane geometry by moving the sample to a spot on the magnetic platen where an out-of-plane component of the field is expected, as per the AFM manual (illustrated in Fig S5e, taken from Asylum Research MFD infinity User manual). Note that this magnitude of the out-of-plane component will likely not match that measured by the Hall probe in the system (quoted in the figure titles in Fig. S5f-h). Again, no changes to the domain structure were seen in the presence of any magnetic field.

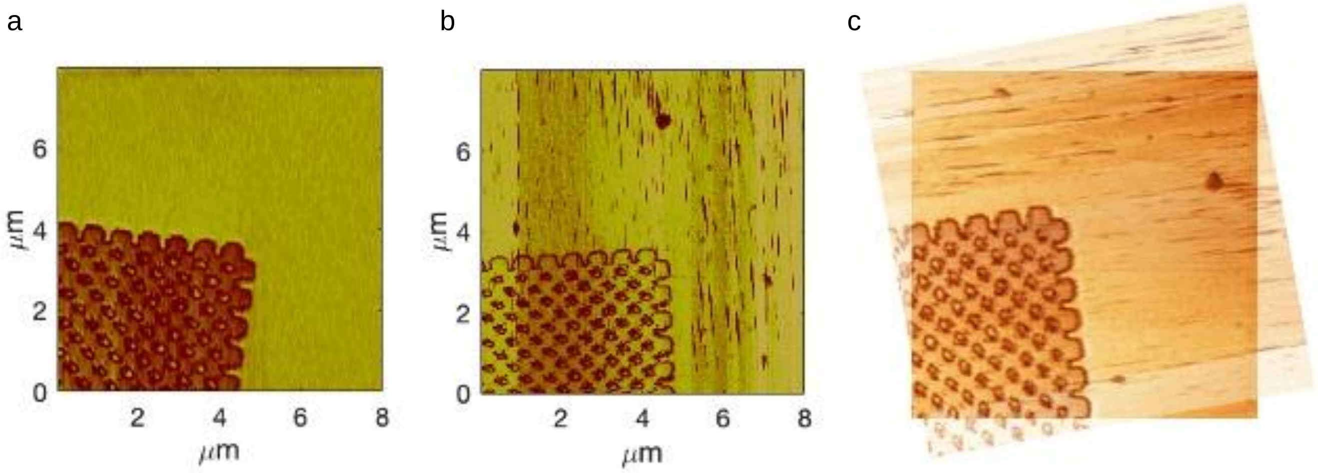

Figure S4 | Magnetic field influence on domain structure. a, PFM amplitude showing the domain structure in LNO before application of a magnetic field cycle similar to that applied in the MR experiment. **b,** PFM amplitude after magnetic field cycle. **c,** An image showing the overlap of PFM pre and post magnetic field cycle. The colour scheme has been changed for clarity.

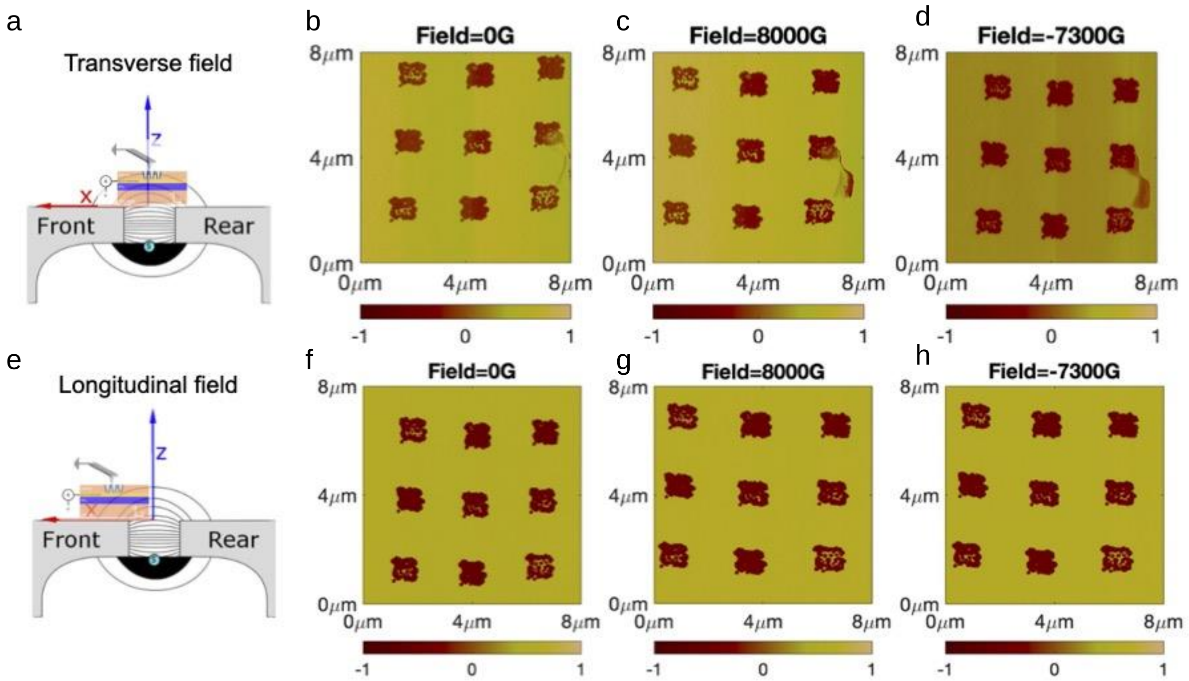

Figure S5 | Magnetic field influence on domain structure in situ. a, Illustration of the field profile from an AFM manual (MFD infinity User manual), illustrating the magnetic field profile for the in-situ AFM magnet. The sample experiences a transverse field. **b-d,** PFM of conducting domain walls at 0G, +8000G and -7300G respectively, in a transverse geometry. **e,** an illustration of the $LiNbO_3$ sample moved slightly, so as to experience a slight longitudinal component of magnetic field. **f-h,** PFM of conducting domain walls at 0G, +8000G and -7300G respectively, in a the longitudinal geometry. Note that here the quoted field (in the subplot titles) is likely higher than the longitudinal field experienced by the sample.

Section S8: IV characteristics of the LNO samples and the possibility of metallicity

We show the current-voltage (IV) characteristics of the LNO samples in Fig. S6. Fig. S6a illustrates the “switching” pulse applied to the fresh LNO surface to induce the domain walls. A 100kOhm resistor was set in series during switching to limit the current through the system. IV performed before and after application of the switching pulse (Fig. S6b) shows clearly a marked and persistent increase in current through the device spanning 4-5 orders of magnitude, confirming the existence of conducting domain walls between the top and bottom electrode. Fig. S6c shows the temperature dependence of IV for a sample with domain walls injected. IV curves were performed at room temperature and then again at various temperatures upon ramping up to a maximum temperature of 80°C (solid lines). IV data was also gathered at the same temperatures on the ramp back down to room temperature (dotted lines). Several things are visible in Fig. S6c:

- Firstly, at a given temperature, the current at a set voltage increases slightly upon application of successive IV loops. This property is well known – the LNO domain wall conductivity state can be changed by application of a sub-coercive bias[2]. This behaviour has been investigated for potential memristive device applications[3].
- Secondly, current clearly increases with increasing temperature. This suggests a semiconducting response; however, we note that these domain wall conductance measurements (along with most others reported in literature) are 2-probe, meaning the response of the electrode contact is convoluted with any real domain wall response.
- Thirdly, there appears to be some hysteresis in the temperature cycle; the current appears to be diminished in the IV curves performed on the ramp “back down” from high temperature. A decay of domain wall conductivity with time is well documented

in LNO domain walls and elevated temperature has been seen to accelerate the decay process. Several mechanisms have been proposed to explain the decay: cross-sectional TEM images have shown the domain wall tilt angle relaxing after some time[2]. This process could, in principle, happen quicker at higher temperatures. It has also been suggested that ion migration occurs, screening the domain wall conductivity. This is supported by the observed fact that conductivity decay appears to be "activated"[4]. We note here again that all conclusions on this matter are also subject to parasitic contact effects, which highlights the need for genuine 4-probe transport measurements on these systems to reveal reliable transport phenomena.

While our IV measurements demonstrate a semiconducting temperature dependence, we reiterate that these measurements, like most performed on domain walls, are 2-probe. The underlying behaviour of the domain wall system could be very different from that suggested in a 2-probe measurement, with the electrode-sample contact playing a dominant role, obscuring the inherent domain wall response. Given the huge change in conductivity demonstrated in these systems, it is interesting to consider, at least theoretically, the possibility of metallic conduction along the domain walls. After all, hints of metallicity have been seen in other domain wall systems (such as barium[5] and lead[6] titanate). Low carrier densities in the LNO domain walls might initially suggest that the Mott criterion for an insulating-to-metal transition should be unlikely, but further consideration shows this is a viable possibility: the conditions needed for a Mott transition are encapsulated in a simple criterion

$$n_c{}^{1/3} a_h{}^* \leq 0.25 \qquad \text{S 48}$$

where n_c is the critical carrier density and $a_h{}^*$ the effective Bohr radius of the electron-centre system. Semiconductors with low effective mass have higher Bohr radii and require a low density of carriers to achieve this transition. If the high mobility that we measure is indicative of a low effective mass, the critical density for metallicity could be low; perhaps low enough to be surpassed by the local change in carrier density at the wall which occurs to screen the bound polarisation charge. Using a rough estimate for the Bohr radius in LNO

$$\boldsymbol{a_h{}^* = \frac{K_{st}\hbar^2}{m^* e^2} = 7 \times 10^{-9} m} \qquad \text{S 49}$$

where, K_{st} is the low frequency static dielectric constant ($K_{st} \sim 84\varepsilon_0$)[7], and m^* is the effective mass of the electron. This is not measured, but an estimate is suggested as $m^* \sim 0.05 m_e$[8]. The critical density for an insulator-metal transition is then:

$$\boldsymbol{n_c \approx 4 \times 10^{16} cm^{-3}} \qquad \text{S 50}$$

It is interesting to note that the domain wall width required to surpass this critical density for metallicity in LNO walls is 0.25nm (using eqn 10 in the main text). While domain wall widths are usually assumed to be in the nanometer range, our estimate is not far off this value. Combined with the rough, order of magnitude nature of the preceding discussion, this analysis suggests metallic transport is a viable possibility for LNO walls. For reference, a wall width of 0.25nm, along with the estimated active carrier density, results in a conductivity of the walls of:

$$\boldsymbol{\sigma_{DW} \approx 2400 \, (\Omega m)^{-1}} \qquad \text{S 51}$$

This is higher than previously reported estimates[4]. However, the domain wall is assumed to be thinner here, and the domain wall inclination angle with respect to the polar axis, which is known to affect wall conduction[9], is also higher in our samples.

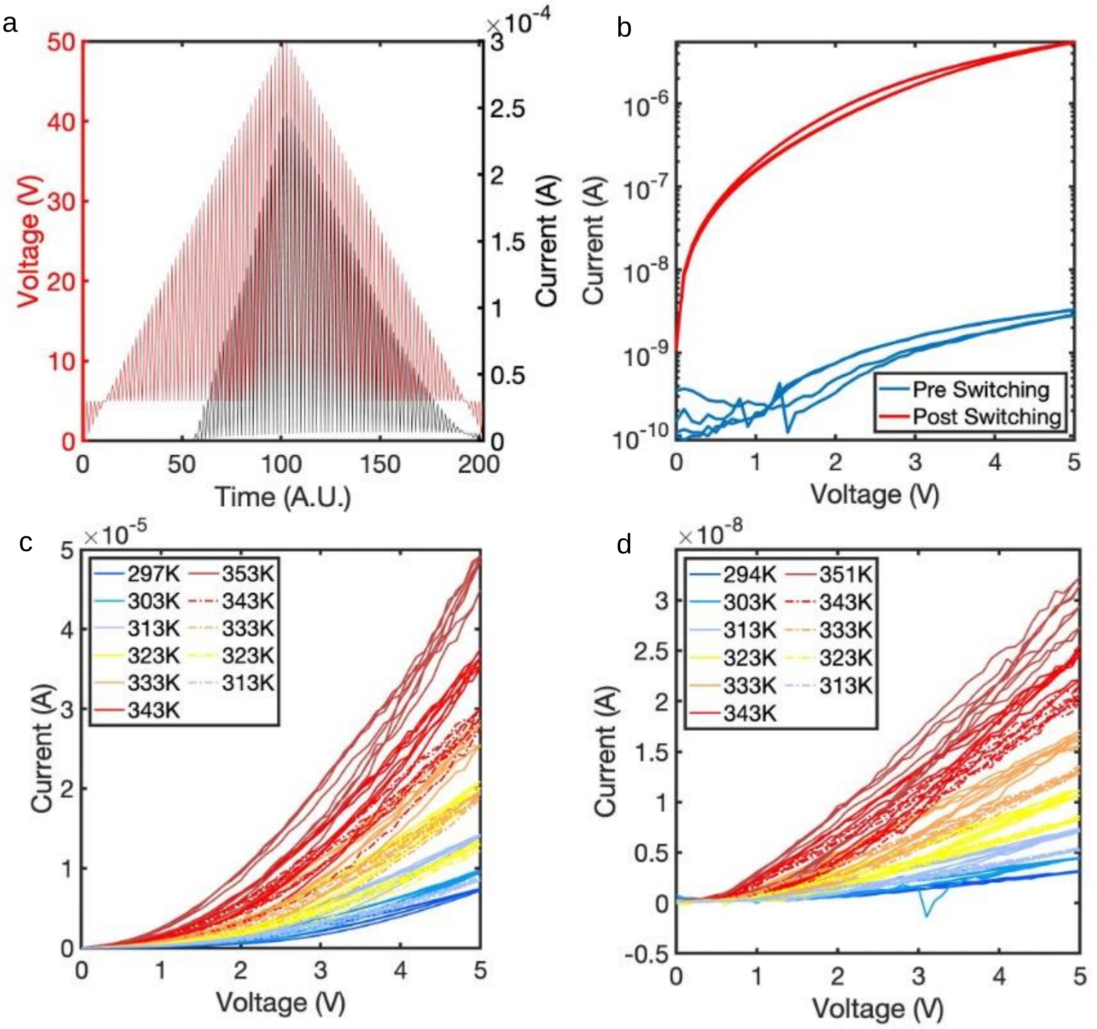

Figure S6 | IV response and temeperature dependent IV response of MR $LiNbO_3$ samples. **a,** The switching pulse, and measured current, applied to the LNO sample to induce domain walls. **b,** IV before and after switching, showing a clear increase in sample conductance **c,** Temperature variation of the IV response of the LNO sample with domain walls induced. Full lines indicate the IV was taken on the ramp up in temperature, dotted lines on the ramp down. **d,** temperature dependent IV of the sample before inducing domain walls, showing the response of the fresh LNO film.

Section S9: MR as a function of field angle

As reported in the main text, we have repeated the MR experiment for a series of magnetic field angles and found no clear variation of the MR (main text Fig. 4b). If anything, the MR increases as the field orientation changes from being parallel to being perpendicular to the conical axes of the Corbino cones. Here we lay out the quantitative derivation for geometric MR with the field applied in the XY plane (hereafter XY-MR). It shows that an XY-MR of similar magnitude to the z-oriented field case is expected (hence the apparent isotropy in the MR response that we measure). The reason MR exists in this field orientation is because the charged "boundaries" that develop do not sustain the full Hall field required to fully cancel Lorentz deflection of carriers. This is a well-known result of current deflection in samples with "intermediate" length to width ratios[10] (Fig. S7c), which are neither long Hall bars ($l/_w > 5$, Fig S7a) nor a sufficiently short Hall bar ($l/_w \ll 1$, shown in Fig. S7b). Note the limiting case $l/_w = 0$ is the Corbino disc.).

First, we discuss the domain wall geometry in detail, estimating the domain wall dimensions, based on microstructural measurements from the main text. This allows us to approximate the effective aspect ratio in our samples. Then, we solve the current density equations for a magnetic field in the x-y plane by Popovic's iterative method, taking care to note than some current components will be partially reduced by a Hall field, with the degree of cancellation governed by a geometry factor. Finally, we derive the form of the XY-MR in terms of this geometry factor. Using estimates for it from literature, we find the geometric XY-MR to be of a similar magnitude to that expected when the magnetic field is parallel to the z-axis, and therefore similar in magnitude to that experimentally measured. This demonstrates that the interpretation of MR as having a geometric origin is fully consistent with all of our experimental observations and that hence the mobility values inferred are likely to be robust. Certainly, there is no need to evoke any mechanism beyond geometric MR to fully explain our data.

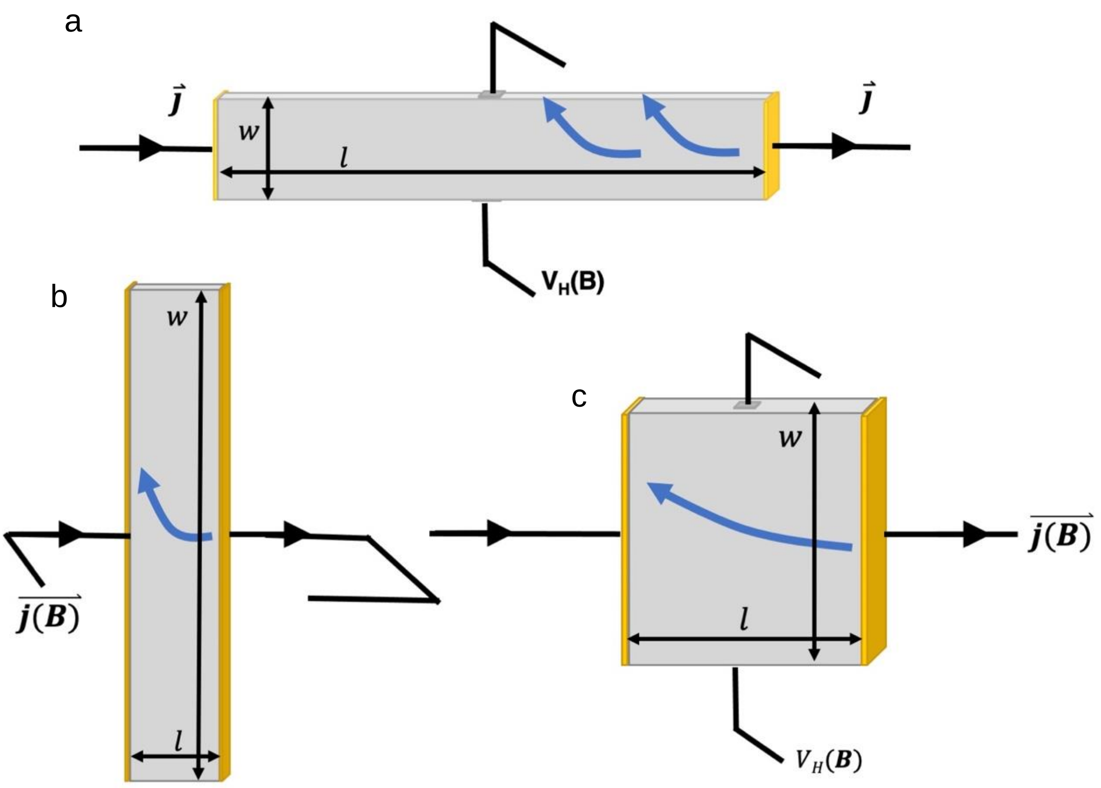

Fig S7| Sample Geometries. a, A standard long Hall bar, with $l/_w > 5$. **b,** A short hall bar, which seeks to maximise the geometric magnetoresistance effect: $l/_w \ll 1$. **c,** An intermediate sample, with $1 < l/_w < 5$. Here, both the Hall effect and geometric MR are present.

<u>Domain wall geometry</u>

Taking the radius of the conical walls at the top electrode as r_1 =150nm, and the wall angle away from the thin film normal as 90-$\theta = 12°$ (where θ is the inclination angle of the walls as defined in the main text) over a film of thickness $t = $ 500nm, we find a bottom radius of

$$r_2 = r_1 - t\tan(90-\theta) = 44nm \qquad \text{S52}$$

as illustrated in Fig. S8a. We can "unwrap" the conducting conical surface to consider it as a 2D truncated sector of a circle, with upper and lower half arc-widths of πr_1 and πr_2, separated by a fixed length (l):

$$l = \frac{t}{\cos(90-\theta)} = 511nm \quad S53$$

as seen in Fig. S8b. Given the shape of the unwrapped cone, the half arc-width of the conductor changes as we traverse from the top to the bottom electrode contacts. Therefore, we define a variable coordinate ρ, which maps the perpendicular distance of any point on the domain wall surface from the top electrode. The half arc-width of the conducting cone at any point ρ is simply half of the circumference of the circular section of the cone at that point. With the help of figure S8a, we can see that the radius of this circular section, as a function of ρ becomes:

$$r(\rho) = r_1 - x' (\rho) = r_1 - \rho \sin(90-\theta) \quad S54$$

The half arc-width ($w(\rho)$) is therefore:

$$w(\rho) = \pi r(\rho) = \pi(r_1 - \rho \sin(90-\theta)) \quad S55$$

Figure S8c shows a plot of ${}^{l}/_{w(\rho)}$ as a function of the normalised coordinate ρ/l, illustrating the aspect ratios involved in our system and how they change along the current path. Half arc-widths have been defined because the Lorentz force associated with XY-MR geometry creates diametrically opposite strips of increased and decreased carrier density (illustrated in figure S9b and S9c). As a result, two symmetrically related regions form which act as short Hall bars, electrically connected in parallel. Ideally, the Hall electric field is azimuthal and so flux lines follow arcs in the 2D representation of the cone, always being perpendicular to the undeflected current direction. The centres of charge density are separated by the width defined in equation S55, such that this half arc-width is the effective width of the Hall bar. We note that the width of the Hall bar varies along the length of the current path.

After ref [11,12], we can also define the "geometrical factor of magnetoresistance", which is the ratio between the measured geometric MR in a sample of intermediate l/w and the geometric magnetoresistance that would be measured in a perfect Corbino disc ($l/w = 0$)

$$g_{MR}({}^{l}/_{w}) = \frac{MR(l/w)}{MR(0)} \quad S52$$

g_{MR} is 1 for a Corbino disc (${}^{l}/_{w} = 0$), and 0 for an infinitely long Hall bar. Physically, g_{MR} tracks how much the shorting of the Hall potential, in samples with intermediate aspect ratios, leads to carrier path bending, and therefore geometric MR. The relative change in transverse resistance can be written as

$$r(B) = \frac{R(B)}{R(0)} = \frac{\sigma(0)}{\sigma(B)}(1 + \mu^2 B^2 g({}^{l}/_{w})) \quad S53$$

Where the first term accounts for physical MR and the second term the geometric MR. For the reasons given in the main text, we assume that the physical MR is insignificant in comparison to the geometric MR term (the usual case when the carrier mobility is reasonably high in comparison to mobility spread). Equations S56 and S57 are valid for $\mu B < 1$. Considering the geometrical MR contribution only, we then find a general magnetoresistance of

$$MR = \frac{R(B)}{R(0)} - 1 = \mu^2 B^2 g({}^{l}/_{w}) \quad S54$$

in intermediate "short-Hall-bar" geometries.

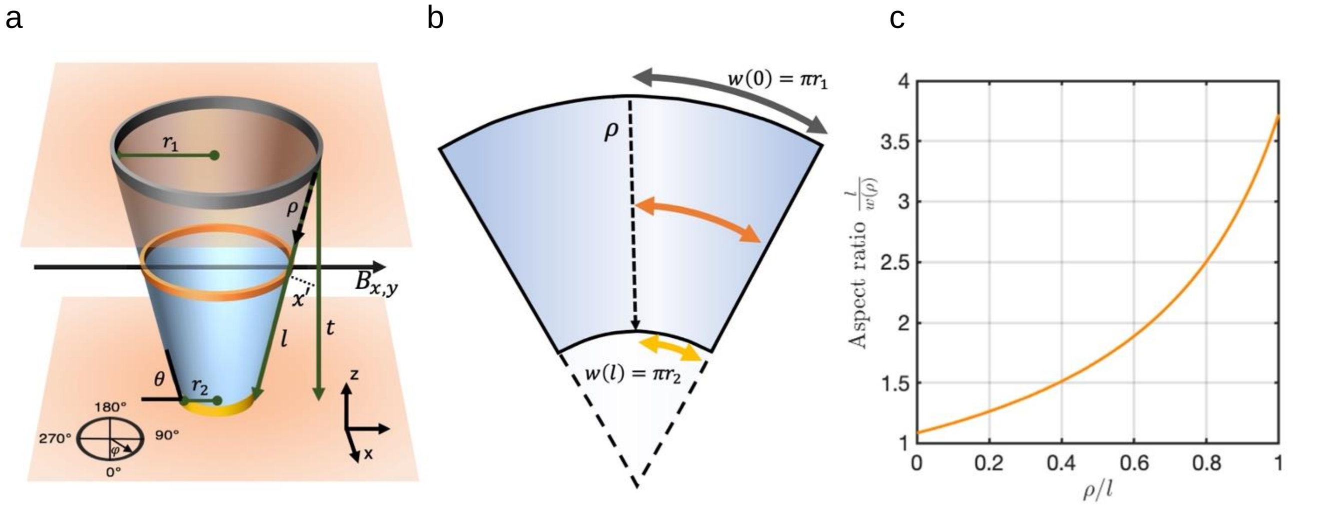

Fig. S8: Domain wall dimensions. a, Schematic of the domain wall cone in the XY-MR configuration with some dimensions labelled. The coloured rings are circular sections of the cone at different depths into the LNO film. **b,** schematic of the cone surface "unwrapped", illustrating the truncated circle sector geometry. Dotted lines represent variables defined in the text above and the solid, double-ended, arrows represent the half arc-widths corresponding to half the circumference of the rings in **a**. **c,** the aspect ratio of the conductor shown in **b**, as a function of normalised length along the current path (ρ/l). The range of aspect ratios are consistent with it being considered as a short Hall bar at all points.

Solution to the current density equations: Here we solve the current density equations for current in a conical geometry when exposed to specific electric and magnetic fields $\boldsymbol{B}$ and $\boldsymbol{E}$. As always, the high symmetry axis of the cone is taken to be aligned along the z-axis, but this time the magnetic field is applied in the x-y plane. We choose the y axis for convenience, but the behaviour is the same as for any other in-plane direction by symmetry. We begin by setting out the field profiles for the solution. In cylindrical base coordinates, the electric and magnetic fields are:

$$\boldsymbol{E} = \begin{pmatrix} E_r \\ E_\varphi \\ E_z \end{pmatrix} = E_0 \begin{pmatrix} -cos\theta \\ 0 \\ -sin\theta \end{pmatrix}; \qquad \text{S55}$$

$$\boldsymbol{B} = \begin{pmatrix} B_r \\ B_\varphi \\ B_z \end{pmatrix} = T_{ij} \begin{pmatrix} B_x \\ B_y \\ B_z \end{pmatrix} = \begin{pmatrix} cos\varphi & sin\varphi & 0 \\ -sin\varphi & cos\varphi & 0 \\ 0 & 0 & 1 \end{pmatrix} \begin{pmatrix} 0 \\ B_0 \\ 0 \end{pmatrix} = \begin{pmatrix} B_0 sin\varphi \\ B_0 cos\varphi \\ 0 \end{pmatrix} \qquad \text{S56}$$

Here, $\boldsymbol{E}$ and $\boldsymbol{B}$ are electric and magnetic field vectors, r, φ and z subscripts represent radial, azimuthal and z components of the vectors, and θ is the inclination angle of the cone away from the horizontal (approx. 78°). T_{ij} is the matrix used to transform vectors from Cartesian space into cylindrical space. φ, when not used in a subscript, represents the azimuthal coordinate.

The calculation of the current density proceeds similarly to that in the Z-MR treatment; the effect of the magnetic field is found by iteratively summing smaller and smaller corrections to the drift current, to successively higher orders of μB. We split each appearing current component into sub-components, which are either embedded in the conducting cone (and allowed to propagate) or directed off the conical surface (to then be cancelled by a Hall potential developed across the width of the domain wall). To do this, we need the unit vector in the direction of the cone surface normal. The electric field, $\widehat{\boldsymbol{E}}$ lies within the cone. Furthermore, since a cone has rotational symmetry, a translation of $\widehat{\boldsymbol{\varphi}}$ also lies within the cone, such that the vector

$$\hat{\boldsymbol{u}} = \hat{\boldsymbol{E}} \times \hat{\boldsymbol{\varphi}} = \begin{pmatrix} sin\theta \\ 0 \\ -cos\theta \end{pmatrix} \quad S61$$

will always be perpendicular to the tangent plane on the cone surface.

We first calculate the drift due to the electric field only:

$$\boldsymbol{j}_0 = \sigma\boldsymbol{E} = \sigma E_0 \begin{pmatrix} -cos\theta \\ 0 \\ -sin\theta \end{pmatrix} \quad S62$$

Then, using the same process as before (equation S19, as used in the Z-MR treatment), we calculate the "first" deflected current term, $\boldsymbol{j}_1$,

$$\boldsymbol{j}_1 = \mu(\boldsymbol{j}_0 \times \boldsymbol{B}) = \sigma\mu E_0 \begin{pmatrix} B_0.sin\theta.cos\varphi \\ -B_0.sin\theta.sin\varphi \\ -B_0.cos\theta.cos\varphi \end{pmatrix} \quad S63$$

In contrast to the Z-MR case, where the first deflected current term is azimuthal (and therefore entirely within the conical surface), this term needs to be split into subcomponents, as the vector does not lie in the conducting cone surface everywhere. First, the "Hall" subcomponent is found by taking the projection of the current along the surface normal ($\hat{\boldsymbol{u}}$), and multiplying by -1 (because a Hall component will act to *oppose* this surface normal current)

$$\boldsymbol{j}_{1,H} = -\frac{\boldsymbol{j}_1 \cdot \hat{\boldsymbol{u}}}{|\hat{\boldsymbol{u}}|^2}\hat{\boldsymbol{u}} = -\sigma\mu E_0 B_0 cos\varphi\, \hat{\boldsymbol{u}} \quad S64$$

Then, the remaining "allowed" current subcomponent is found by adding the total current component and the Hall subcomponent corrections:

$$\boldsymbol{j}_{1,All} = \boldsymbol{j}_1 + \boldsymbol{j}_{1,H} = \sigma\mu E_0 \begin{pmatrix} 0 \\ -B_0.sin\theta.sin\varphi \\ 0 \end{pmatrix} \quad S65$$

We find that only an azimuthal term now survives. Inspecting equation S65, we see that the deflection of carriers now depends on the azimuthal coordinate, φ. In other words, the magnitude and sense of the deflection depends on the angular location on the conical surface. We can rationalize this with the help of figure S9a. At the points in the cone corresponding to $\varphi = 0°$and $180°$, the deflection takes us directly off the cone, and so should be fully compensated by a Hall field across the domain wall width. Conversely, this deflection remains fully within the cone at points $\varphi = 90°, 270°$, leading to maximal allowed azimuthal current components at these points. $\boldsymbol{j}_{1,All}$ is illustrated by red arrows in fig S9a. This explains the $sin\varphi$ dependence in the first current component.

In contrast to the Z-MR treatment (where both the original system and the perturbing magnetic field had rotational symmetry about the z axis), we now have a situation where the rotational symmetry is broken by the magnetic field. Therefore, we might expect that properties such as carrier density, current density etc will vary with azimuthal angle φ. If we look at the deflection implied by equation S65, we find that there is a preference for carriers to deflect towards one side of the cone (Fig. S9a), which should lead to the formation of a new Hall potential (Fig. S9b) distinct from that present across the width of the domain wall itself.

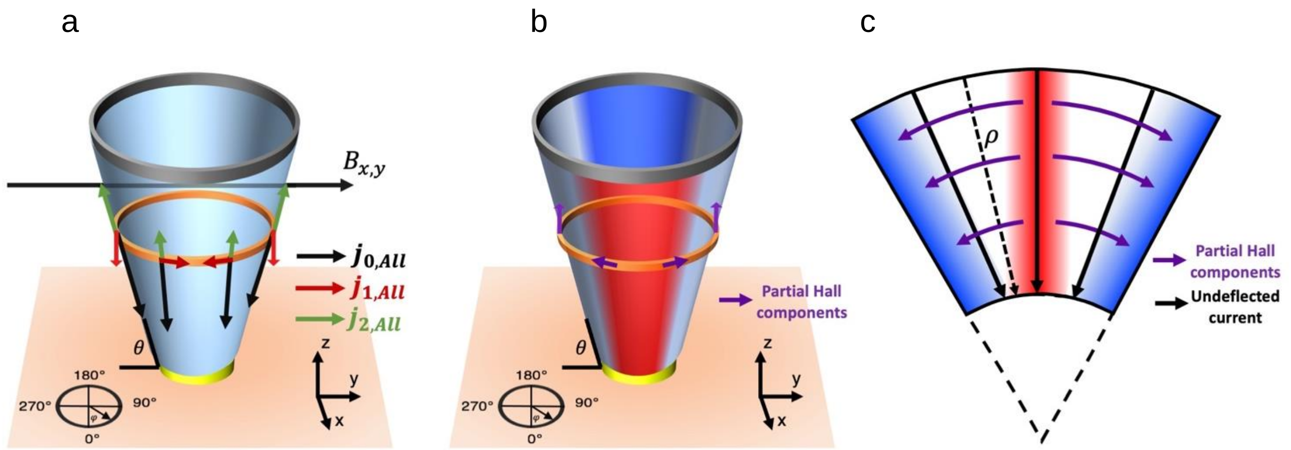

Fig S9: Current components with magnetic field in the x-y plane a, Current components obtained from the iterative solution. **b,** The building of a Hall potential across the conducting cone itself. Red and blue shaded areas indicate the accumulation/depletion of carriers, and purple arrows represent the effective current density which would result from the associated partial Hall potential difference developed across this effective pair of short Hall bars. **c,** the unwrapped cone, with red and blue regions highlighting the building of the Hall potential across the half arc widths. Purple arrows represent the azimuthal Hall flux lines, and the black solid arrows represent the undeflected current. The dotted black line represents the variable distance ρ.

We need to assess the degree to which this new Hall potential will cancel the Lorentz deflection of carriers for this component. As mentioned above, this comes down to the geometry of the situation; specifically, the length-to-width ratio of the current channel, and the term $g_{MR}(^{l}/_{w})$ in equation S56. Here, length is the distance between current carrying electrodes, and width is the arc length distance between the strips where charge accumulation / denudation occurs (red and blue strips in figure S9b). Figure S9c shows the cone unwrapped into a truncated circular sector, illustrating the Hall potential more clearly. As we can see, the red and blue strips are separated by half the circumference of the circular section of the cone at all points ρ. The Hall electric field will act azimuthally, along the circular arcs of the conducting cone (purple arrows), to counteract the deflection of current due to the magnetic part of the Lorentz force. This cancellation is not total, however, because the geometry reflects that of a short Hall bar. We therefore assume that the new fractional current, allowed in the $\hat{\boldsymbol{\varphi}}$ direction, is simply the full allowed current density, multiplied by the geometry factor g_{MR}. Essentially, Hall fields now act to cancel all of the $\hat{\boldsymbol{u}}$ oriented current, and some fraction of the $\hat{\boldsymbol{\varphi}}$ oriented current, where the fraction is determined by g_{MR}:

$$\boldsymbol{j}_{1,All} = \boldsymbol{j}_1 + \boldsymbol{j}_{1,H} = \boldsymbol{j}_1 - \frac{\boldsymbol{j_1} \cdot \hat{\boldsymbol{u}}}{|\hat{\boldsymbol{u}}|^2}\hat{\boldsymbol{u}} - G_H \frac{\boldsymbol{j_1} \cdot \hat{\boldsymbol{\varphi}}}{|\hat{\boldsymbol{\varphi}}|^2}\hat{\boldsymbol{\varphi}} \qquad \text{S66}$$

Here, G_H determines the amount of current along the $\hat{\boldsymbol{\theta}}$ direction which is cancelled by the partial Hall potential, such that

$$1 - G_H = g_{MR} \qquad \text{S67}$$

Evaluating this, we find a current density

$$\boldsymbol{j}_{1,All} = \sigma\mu E_0 B_0 sin\theta\ sin\varphi(1 - G_H)\begin{pmatrix} 0 \\ -1 \\ 0 \end{pmatrix} \qquad \text{S68}$$

Continuing the iterative approach, the next current component is:

$$\boldsymbol{j}_2 = \mu(\boldsymbol{j}_{1,All} \times \boldsymbol{B}) = \sigma E_0 \mu^2 B_0^2 (1 - G_H) \begin{pmatrix} 0 \\ 0 \\ sin\ \varphi^2\ sin\ \theta \end{pmatrix} \qquad \text{S69}$$

Again, we project this component along $\hat{\boldsymbol{u}}$ and $\hat{\boldsymbol{\varphi}}$ to find the appropriate Hall fields and resulting allowed current:

$$\boldsymbol{j}_{2,All} = \boldsymbol{j}_2 - \frac{\boldsymbol{j}_2 \cdot \hat{\boldsymbol{u}}}{|\hat{\boldsymbol{u}}|^2}\hat{\boldsymbol{u}} - G_H \frac{\boldsymbol{j}_2 \cdot \hat{\boldsymbol{\varphi}}}{|\hat{\boldsymbol{\varphi}}|^2}\hat{\boldsymbol{\varphi}} \qquad \text{S70}$$

Explicitly:

$$\boldsymbol{j}_{2,All} = \sigma E_0 \mu^2 B_0^2 (1 - G_H) \begin{pmatrix} 0 \\ 0 \\ sin\ \varphi^2\ sin\ \theta \end{pmatrix} - (-\mu^2 B_0^2 \sigma E_0 sin^2\varphi\ sin\ \theta\ cos\theta)\ (1 - G_H) \begin{pmatrix} sin\theta \\ 0 \\ -cos\theta \end{pmatrix} - G_H \begin{pmatrix} 0 \\ 0 \\ 0 \end{pmatrix} \qquad \text{S71}$$

Which we can write in terms of the external electric field

$$\boldsymbol{j}_{2,All} = -\mu^2 B_0^2 \sigma E_0 sin^2\varphi sin^2\theta\ (1 - G_H) \begin{pmatrix} -cos\theta \\ 0 \\ -sin\theta \end{pmatrix} = -\alpha\sigma\boldsymbol{E} \qquad \text{S72}$$

where

$$\alpha = \mu^2 {B_0}^2 sin^2\theta . sin^2\varphi (1 - G_H) \qquad \text{S73}$$

Next:

$$\boldsymbol{j}_3 = \mu(\boldsymbol{j}_{2,All} \times \boldsymbol{B}) = -\mu^3 B_0^3 \sigma E_0 sin^2\varphi sin^2\theta\ (1 - G_H) \begin{pmatrix} sin\theta . cos\varphi \\ -sin\theta . sin\varphi \\ -cos\theta . cos\varphi \end{pmatrix} \qquad \text{S74}$$

and again, we find the allowed component:

$$\boldsymbol{j}_{3,All} = \boldsymbol{j}_3 - \frac{\boldsymbol{j}_3 \cdot \hat{\boldsymbol{u}}}{|\hat{\boldsymbol{u}}|^2}\hat{\boldsymbol{u}} - G_H \frac{\boldsymbol{j}_3 \cdot \hat{\boldsymbol{\varphi}}}{|\hat{\boldsymbol{\varphi}}|^2}\hat{\boldsymbol{\varphi}} = \mu^3 B_0^3 \sigma E_0 sin^3\varphi sin^3\theta\ (1 - G_H)^2 \begin{pmatrix} 0 \\ 1 \\ 0 \end{pmatrix} = \alpha\sigma\mu E_0 B_0 sin\theta\ sin\varphi (1 - G_H) \begin{pmatrix} 0 \\ 1 \\ 0 \end{pmatrix} \qquad \text{S75}$$

The next component is

$$\boldsymbol{j}_4 = \mu^4 B_0^4 \sigma E_0 sin^3\varphi sin^3\theta\ (1 - G_H)^2 \begin{pmatrix} 0 \\ 0 \\ -sin\varphi \end{pmatrix} \qquad \text{S76}$$

giving the allowed components:

$$\boldsymbol{j}_{4,All} = \mu^4 B_0^4 \sigma E_0 sin^4\varphi sin^4\theta\ (1 - G_H)^2 \begin{pmatrix} -cos\theta \\ 0 \\ -sin\theta \end{pmatrix} = \alpha^2\sigma\boldsymbol{E} \qquad \text{S77}$$

One more current iteration allows us to see the pattern clearly:

$$\boldsymbol{j}_5 = \mu(\boldsymbol{j}_{4,All} \times \boldsymbol{B}) = \mu^5 B_0^5 \sigma E_0 sin^4\varphi sin^4\theta\ (1 - G_H)^2 \begin{pmatrix} sin\theta . cos\varphi \\ -sin\theta . sin\varphi \\ -cos\theta . cos\varphi \end{pmatrix} \qquad \text{S78}$$

and

$$\boldsymbol{j}_{5,All} = -\mu^5 B_0^5 \sigma E_0 sin^5\varphi sin^5\theta\,(1-G_H)^3 \begin{pmatrix}0\\1\\0\end{pmatrix} = -\alpha^2\sigma\mu E_0 B_0 sin\theta\; sin\varphi(1-G_H)\begin{pmatrix}0\\1\\0\end{pmatrix} \qquad \text{S79}$$

Summing the allowed components, we find a current density of

$$\boldsymbol{j}_{total} = \sum_{\boldsymbol{n=0}}^{\infty} \boldsymbol{j}_{\boldsymbol{n},All} = (1-\alpha+\alpha^2-\alpha^3 \ldots)\sigma\left(\boldsymbol{E} + E_0\begin{pmatrix}0\\-\mu B_0 sin\theta\; sin\varphi(1-G_H)\\0\end{pmatrix}\right) \qquad \text{S80}$$

Which generates a full solution:

$$\boldsymbol{j}_{total} = \frac{1}{1+\alpha}\left(\sigma\boldsymbol{E} + \sigma E_0\begin{pmatrix}0\\-\mu B_0 sin\theta\; sin\varphi(1-G_H)\\0\end{pmatrix}\right) \qquad \text{S81}$$

In a similar way to the z-directed magnetic field case, our magnetoresistance is given by

$$MR(\varphi) = \alpha = \mu^2 {B_0}^2 sin^2\theta . sin^2\varphi(1-G_H) \qquad \text{S82}$$

Integrating over the entire cone, to remove the φ dependence and obtain MR, in terms of current (rather than current density)

$$MR = \frac{1}{2\pi}\int_0^{2\pi} \mu^2 {B_0}^2 sin^2\theta . sin^2\varphi(1-G_H)d\varphi = \frac{\mu^2 {B_0}^2 sin^2\theta g_{MR}}{2} \qquad \text{S83}$$

where the substitution in S67 has been made.

We can turn to literature, in order to estimate g_{MR}. In rectangular plates, Lippmann and Kuhrt[11] first derived g_{MR} for the full aspect ratio-Hall angle parameter space. In regions where both the Hall angle is small ($\tan(\Theta) = \mu B \approx \Theta \ll 1$) and where $\frac{l}{w}$ >1, equations S56-S58 are valid, with the geometric factor of magnetoresistance approximated by

$$g\left({}^{l}/_{w}\right) = \frac{14}{\pi^3} S_3 \frac{w}{l} \qquad \text{S84}$$

where S_3 is a constant approximately equal to 1.2. Using this form of the geometric factor, the XY-MR can be calculated across the entire range of aspect ratios that our domain walls exhibit and the average geometric factor used as a measure of how our system will behave. This is evaluated by integrating the function in equation S84 with respect to $\gamma = l/w$ between the limiting aspect ratios demonstrated in figure S8c ($\gamma_{min} = 1.09, \gamma_{max} = 3.72$). We find the average geometric factor for our system is:

$$g\left({}^{l}/_{w}\right)_{ave} = \frac{1}{\gamma_{max}-\gamma_{min}}\int_{\gamma_{min}}^{\gamma_{max}} \frac{14}{\pi^3} S_3 \frac{1}{\gamma}\; d(\gamma) = \frac{1}{2.63}\frac{14}{\pi^3} S_3\; \ln\left(\frac{3.72}{1.09}\right) = 0.25 \qquad \text{S85}$$

We therefore find a magnetoresistance of:

$$MR_{xy} = \frac{0.25\,\mu^2 {B_0}^2 sin^2\theta}{2} \qquad \text{S86}$$

From our measurements presented in the main text, MR_{xy}~0.4×10^{-2}at 1T, but we think this is slightly suppressed by series contact resistance (MR_{xy}~0.6×10^{-2} at 1T might be closer to the true value, as this was the Z-MR obtained with lower resistance contacts). Using equation S86, to estimate

the carrier mobility implied, gives (to 1 significant figure) a value of 2,000cm^{2}V^{-1}s^{-1}. Another way of probing the physics implied is to use the mobility value estimated from our Z-MR measurements (3,700cm^{2}V^{-1}s^{-1}) to see how large the MR_{xy} could be. Doing this, we find values in the order of 1% at 1T - more than enough to explain the experimentally measured effect.

It should be noted that these XY-MR estimates are sensitive to the values of conical domain dimensions used and to the accuracy of the g_{MR} value determined by Lippmann and Kuhrt [11]; nevertheless, our analysis shows clearly that the measured XY-MR can be fully explained as being geometric in origin. Moreover, the carrier mobilities thereby inferred from the XY-MR measurements are of the same order of magnitude as those found through Z-MR measurements.

Section S10: Domain wall current

The conduction AFM images in figure 1 in the main text show significant smearing of the current across the nanodomain centre. This is a well-known resolution problem with cAFM, We show below that the current at LNO domain walls is certainly confined to the walls, as opposed to the centre of nanodomains. Figure S10 **a-d** shows dual AC resonance tracking PFM (DART-PFM) of LNO domain walls. These domains are poled using an AFM tip top electrode, with various pulse duration (increasing along the x-axis) and increasing pulse magnitude (along the -y-axis). S10**e,f** show conducting AFM in the same region, showing clearly that the enhanced conduction is confined to the domain walls.

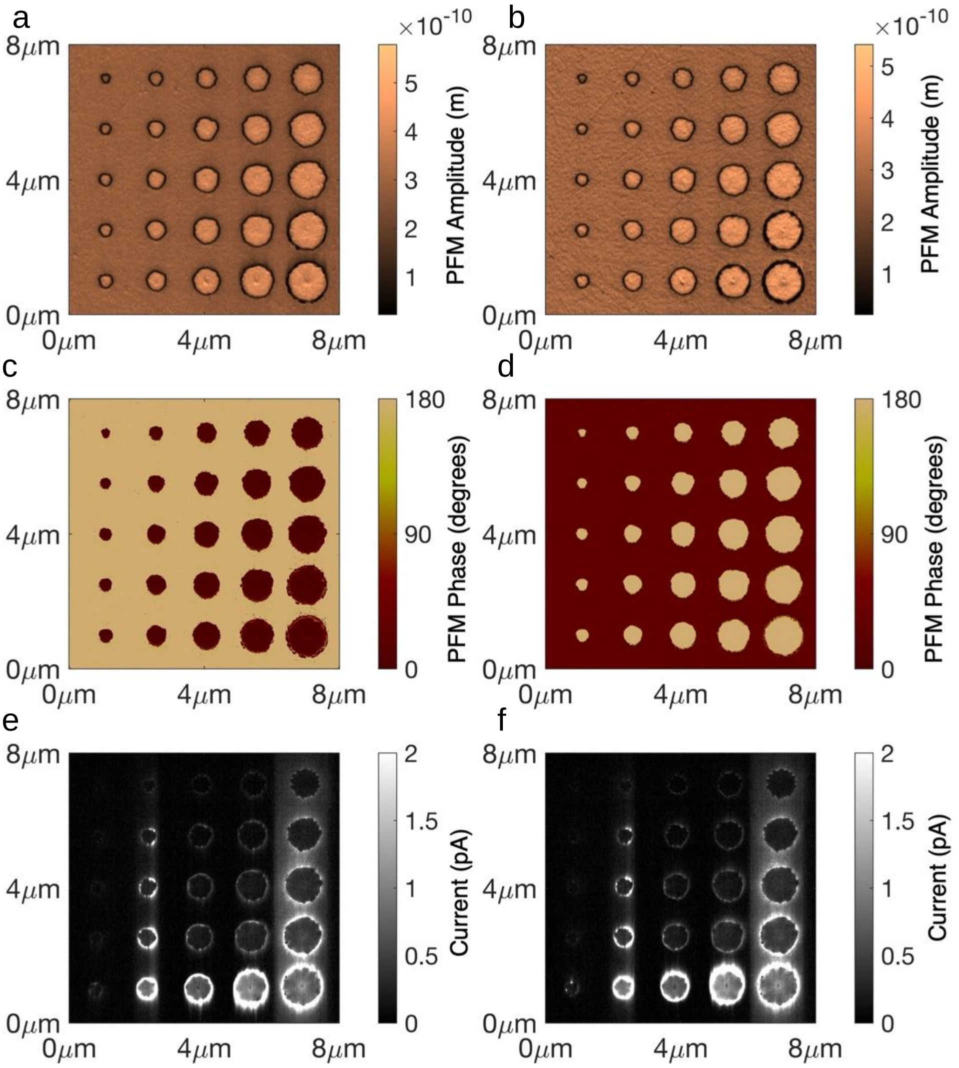

Figure S10 | Domain wall currents. **a, b**, PFM amplitude and **c, d**, PFM phase, taken in dual resonance tracking PFM (DART-PFM) mode. These domains are poled using an AFM tip top electrode, with various pulse duration (increasing along the x-axis) and increasing pulse magnitude (along the -y-axis). **e, f** Conduction-AFM from the same regions as **a-d**.

References

1. Popovic, R. S. *Hall Effect Devices*. *Hall Effect Devices, Second Edition* (CRC Press, 2003).
2. Lu, H. *et al.* Electrical Tunability of Domain Wall Conductivity in LiNbO 3 Thin Films. *Adv. Mater.* **31**, 1902890 (2019).
3. McConville, J. P. V. *et al.* Ferroelectric Domain Wall Memristor. *Adv. Funct. Mater.* **30**, 2000109 (2020).
4. Werner, C. S. *et al.* Large and accessible conductivity of charged domain walls in lithium niobate. *Sci. Rep.* **7**, 9862 (2017).
5. Sluka, T., Tagantsev, A. K., Bednyakov, P. & Setter, N. Free-electron gas at charged domain walls in insulating BaTiO3. *Nat. Commun.* **4**, 1808 (2013).
6. Stolichnov, I. *et al.* Bent Ferroelectric Domain Walls as Reconfigurable Metallic-Like Channels. *Nano Lett.* **15**, 8049–8055 (2015).
7. Weis, R. S. & Gaylord, T. K. Lithium niobate: Summary of physical properties and crystal structure. *Appl. Phys. A Solids Surfaces* **37**, 191–203 (1985).
8. Eliseev, E. A., Morozovska, A. N., Svechnikov, G. S., Gopalan, V. & Shur, V. Y. Static

conductivity of charged domain walls in uniaxial ferroelectric semiconductors. *Phys. Rev. B* **83**, 235313 (2011).
9. Godau, C., Kämpfe, T., Thiessen, A., Eng, L. M. & Haußmann, A. Enhancing the Domain Wall Conductivity in Lithium Niobate Single Crystals. *ACS Nano* **11**, 4816–4824 (2017).
10. Jervis, T. R. & Johnson, E. F. Geometrical magnetoresistance and hall mobility in gunn effect devices. *Solid State Electron.* **13**, 181–189 (1970).
11. Lippmann, H. J. & Kuhrt, F. The Geometrical Influence on the Transverse Magnetoresistance for Rectangular Semiconductor Hall Plates. *Zeitschrift für Naturforsch.* **13a**, 462–474 (1958).
12. Heremans, J. Solid state magnetic field sensors and applications. *J. Phys. D. Appl. Phys.* **26**, 1149–1168 (1993).
